# microRNAs with AAGUGC seed motif constitute an integral part of an oncogenic signaling network

**DOI:** 10.1038/onc.2016.242

**Published:** 2016-08-01

**Authors:** Y Zhou, O Frings, R M Branca, J Boekel, C le Sage, E Fredlund, R Agami, L M Orre

**Affiliations:** 1 Department of Oncology-Pathology, Science for Life Laboratory, Karolinska Institutet, Stockholm, Sweden; 2 Bioinformatics Infrastructure for Life Sciences (BILS), Stockholm, Sweden; 3 Division of Biological Stress Response, The Netherlands Cancer Institute, Amsterdam, The Netherlands

## Abstract

microRNA (miRNA) dysregulation is a common feature of cancer cells, but the complex roles of miRNAs in cancer are not fully elucidated. Here, we used functional genomics to identify oncogenic miRNAs in non-small cell lung cancer and evaluate their impact on response to epidermal growth factor (EGFR)-targeting therapy. Our data demonstrate that miRNAs with an AAGUGC motif in their seed sequence increase both cancer cell proliferation and sensitivity to EGFR inhibitors. Global transcriptomics, proteomics and target prediction resulted in the identification of several tumor suppressors involved in the G1/S transition as AAGUGC-miRNA targets. The clinical implications of our findings were evaluated by analysis of AAGUGC-miRNA expression in multiple cancer types, supporting the link between this miRNA seed family, their tumor suppressor targets and cancer cell proliferation. In conclusion, we propose the AAGUGC seed motif as an oncomotif and that oncomotif-miRNAs promote cancer cell proliferation. These findings have potential therapeutic implications, especially in selecting patients for EGFR-targeting therapy.

## Introduction

One of the most studied areas in cancer biology is related to cancer growth and oncogenic drivers of cancer cell proliferation. In addition to expanding the general knowledge of cancer biology, a major reason for this focus is that oncogenic drivers are tempting targets for anti-cancer therapy. The majority of targeted cancer therapies in use today or in clinical studies are inhibitors of proteins that increase cancer cell proliferation as exemplified by epidermal growth factor tyrosine kinase inhibitors (EGFR-TKIs) in non-small cell lung cancer (NSCLC) or ERBB2 inhibitors in breast cancer. Clinical studies have shown us that this type of targeted therapy is only efficient against tumors that rely on the targeted protein for proliferation. Furthermore, it is likely that several parallel pathways/networks need to be targeted simultaneously to achieve long-term efficacy and combat resistance development. To predict treatment response and select targeted therapies, it is therefore of great importance to fully understand the signaling networks that drive cancer cell proliferation.

In spite of massive research efforts, knowledge of specific oncogenic drivers is still lacking in a large part of cancer cases.^1, 2, 3^ One of the reasons for this incomplete knowledge of oncogenic pro-growth signaling is the multiple levels of regulation used by the cancer cells, that is, epigenetic, transcriptional, translational and posttranslational regulation. To complete the current fragmentary picture, additional levels will be needed in the analysis, such as protein-level analysis by mass spectrometry (MS)-based proteomics. These methods are currently reaching sufficient analytical depth and throughput to be integrated in systems biology analysis as shown by us^4^ and others,^5^ and will certainly further improve our knowledge of cancer biology.

One important level of regulation used in cells is posttranscriptional regulation by microRNAs (miRNAs). miRNAs are small, non-coding RNAs that repress gene expression through base pairing between the miRNA seed sequence (5' nucleotides 1–8) and 3' untranslated regions (3'UTRs) of mRNAs, causing mRNA degradation, translation inhibition or both.^6^ Each miRNA can target hundreds of different mRNAs, and it has been estimated that the 1000–1500 different miRNAs expressed in the human genome collectively have the capacity to repress more than 50% of all protein-coding genes.^7^ A huge body of evidence supports the importance of miRNA deregulation in cancer, and both overexpression of cancer-promoting miRNAs (oncomiRs) and loss of cancer-inhibiting miRNAs (tumor suppressor (TS) miRs) are common.^8, 9^ However, the complex target spectrum and biology of miRNAs complicates the interpretation of data and consequently, even when measured, miRNA deregulation is often neglected when presenting the oncogenic drivers in cancer landscape publications.

In this study, we used a functional genomics approach to identify potentially oncogenic miRNAs in NSCLC. Our analysis indicated that expression of miRNAs with an AAGUGC motif in the seed sequence resulted in increased cellular proliferation, which, interestingly, was accompanied by increased sensitivity to EGFR-TKI inhibitors. Molecular profiling of the effects of AAGUGC-miRNA expression at the mRNA and protein level, as well as miRNA target prediction analysis resulted in a large number of potential AAGUGC-miRNA targets. Among these targets were several well-known TSs, explaining the proliferation promoting activities of AAGUGC-miRNAs. Expression of AAGUGC-miRNAs and targets were then evaluated in a number of different cancer types using public domain data. Collectively, our data have led us to suggest the AAGUGC seed sequence motif in miRNAs as an 'oncomotif' and, in addition, a model where oncomotif-miRNAs are an integral part of a signaling network that drives cancer cell proliferation.

## Results

### miRNAs with an AAGUGC motif in their seed sequence increase proliferation and EGFR-TKI sensitivity in NSCLC cells

**Figure 1:**
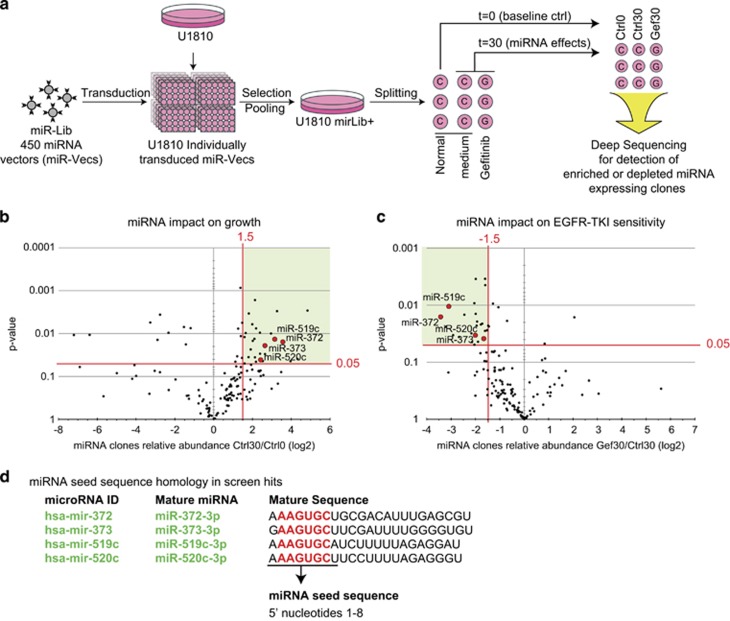
miRNAs with an AAGUGC motif increase proliferation and EGFR-TKI sensitivity. (**a**) Overview of the functional genomics screen for potentially oncogenic miRNAs with an impact on EGFR-TKI sensitivity. A library of different miRNA expression vectors (miR-Lib) was used for individual transduction into U1810 NSCLC cells. After culturing with or without EGFR-TKI (gefitinib, 10uM), miRNA inserts from all samples were recovered by PCR and enrichment or depletion of specific miRNA-expressing clones was determined by deep sequencing. (**b**) Analysis of the relative abundance of recovered miRNA inserts comparing baseline control cells and cells grown in normal medium for 30 days. Volcano plot indicates miRNAs with a positive impact on proliferation. Red lines indicate arbitrarily chosen cutoffs for miRNA clones enriched after 30 days in culture (Ctrl30/Ctrl0, 1.5 log2, *P*<0.05). (**c**) Analysis of the relative abundance of recovered miRNA inserts comparing cells grown in normal medium or gefitinib for 30 days. Volcano plot indicates miRNAs conferring EGFR-TKI sensitivity (miRNA clones depleted after treatment with gefitinib, Gef30/Ctrl30, −1.5 log2, *P*<0.05). (**d**) Four miRNAs enriched after long-term culturing and depleted after treatment with gefitinib share the same AAGUGC motif in the miRNA seed sequence (also indicated in **b** and **c**).

To identify potentially oncogenic miRNAs in NSCLC and to investigate their impact on EGFR-TKI response, a functional genomics screen was performed (Figure 1a). In brief, a library of miRNA expression vectors (*n*=~450)^10^ was individually transduced into the NSCLC cell line U1810, and after selection of stable miRNA expressing cells (~80% of library vectors yielded stable clones), cells were pooled and split into nine cultures. Twenty-four hours after seeding, triplicate untreated cultures were harvested for use as a baseline reference. Triplicate cultures were then treated with an EGFR-TKI (gefitinib) or left untreated for a period of 30 days. At the end of the experiment, altered relative abundances of miRNA-expressing populations between the three conditions (Ctrl0, Ctrl30 and Gef30) were evaluated by deep sequencing (Supplementary Table 1).

The main focus of our analysis was on miRNA-expressing clones that were enriched after 30 days of culture in normal medium (indicating increased proliferation, Figure 1b), and miRNA clones that were depleted after EGFR-TKI treatment (indicating increased EGFR-TKI sensitivity, Figure 1c). We arbitrarily considered only miR-Vecs that had been sequenced at least 300 times in all three replicates of one condition, resulting in 140 unique miR-Vec miRNAs remaining for the enrichment/depletion analysis. Interestingly, several (11) of the miR-Vec miRNAs that increased proliferation also increased EGFR-TKI sensitivity. In addition, a group of these miR-Vecs contain miRNAs (hsa-mir-372, hsa-mir-373, hsa-mir-519c and hsa-mir-520c) with strong seed sequence homology, as they all contain an AAGUGC motif in the 5' end (nt 1–8) of the mature miRNA (Figure 1d). As previous studies have shown oncogenic potential for miRNAs with an AAGUGC motif in their seed sequence, we decided to investigate this group of miRNAs further.

**Figure 2:**
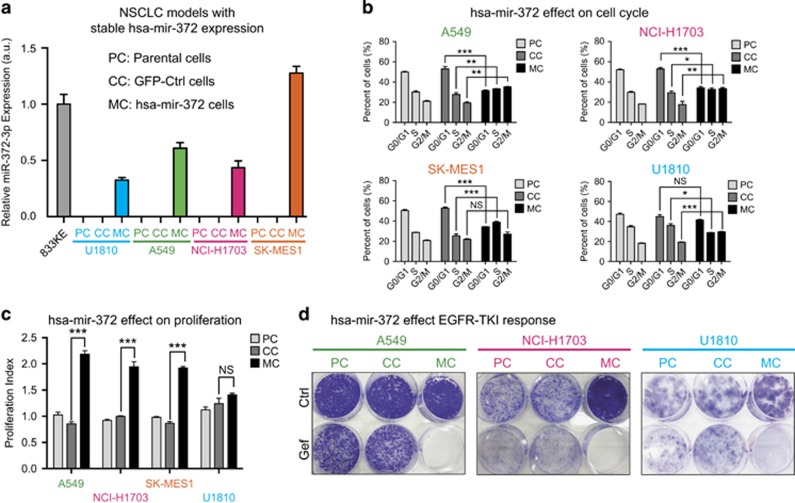
hsa-mir-372 expression increase proliferation and EGFR-TKI sensitivity. (**a**) Relative expression of miR-372-3p in four different NSCLC cell line models stably transduced for hsa-mir-372 expression as measured by RT–qPCR. As a reference, endogenous expression of miR-372-3p in the testicular germ cell cancer cell line 833KE was also measured. (**b**) Analysis of hsa-mir-372 impact on cell cycle distribution indicates a general trend where hsa-mir-372 expression results in a decrease in the G0/G1 population, and an increase of the S and G2/M populations. (**c**) In three out of four cell line models, hsa-mir-372 expression resulted in an increased proliferation index. (**d**) Expression of hsa-mir-372 results in increased sensitivity to EGFR-targeting therapy (gefitinib, 10 uM) as shown for A549, NCI-H1703 and U1810 cells using clonogenic assay. Throughout the figure, bars represent mean values of three replicate experiments, error bars indicate standard deviation, *P*-value calculation was performed by *t*-test and reported as follows: *P*>0.05(NS), *P*<0.05(*), *P*<0.01(**) and *P*<0.001(***).

To validate the impact of AAGUGC-miRNA expression on cell proliferation and EGFR-TKI sensitivity, four different lung cancer cell lines (U1810, A549, NCI-H1703 and SK-MES-1) were stably transduced with a hsa-mir-372 (hereafter refered to as miR-372) expression vector or a control vector. Stably transduced cells were fluorescence-activated cell-sorted based on green fluorescent protein (GFP) expression from the miR-372 and control vectors. qPCR analysis of miR-372-3p expression in all parental, control and miR-372 transduced cells demonstrated the absence of miR-372-3p in all parental and control cells and clear expression in all four miR-372 transduced cell lines (Figure 2a). As a reference, we also measured miR-372-3p expression level in a testicular germ cell tumor cell line (833KE) with previously shown endogenous expression of miR-372-3p,^10^ indicating that the miR-372 expression in our NSCLC models was physiologically relevant. Flow cytometry analysis showed that miR-372 expression resulted in altered cell cycle distribution in all four miR-372 models, with a general trend of a decrease in the G0/G1 population, and an increase of the S and G2/M populations (Figure 2b). In concordance with the results from the functional genomics screen, miR-372 expression resulted in an increased proliferation index, defined as the ratio between S/G2/M cells and G0/G1 cells in three of the miR-372 models (A549, NCI-H1703 and SK-MES-1, Figure 2c). The impact of miR-372 expression on EGFR-TKI sensitivity was evaluated using clonogenic survival assay in U1810, A549 and NCI-H1703 cell line models (Figure 2d). Strikingly, in all three cell line models, EGFR inhibition using gefitinib resulted in a complete loss of colonies formed by miR-372 expressing cells, while only causing a modest decrease in colony size in parental and control cells. The results from the clonogenic assay therefore fully supported the results from the functional genomics screen. In summary, our functional genomics screen indicated that expression of miR-372 and other AAGUGC-containing miRNAs in lung cancer cells results in increased proliferation and increased sensitivity to EGFR inhibitors. Further, these results were confirmed for miR-372 in several NSCLC models stably expressing miR-372.

### Global target analysis of the AAGUGC-miRNA miR-372-3p

**Figure 3:**
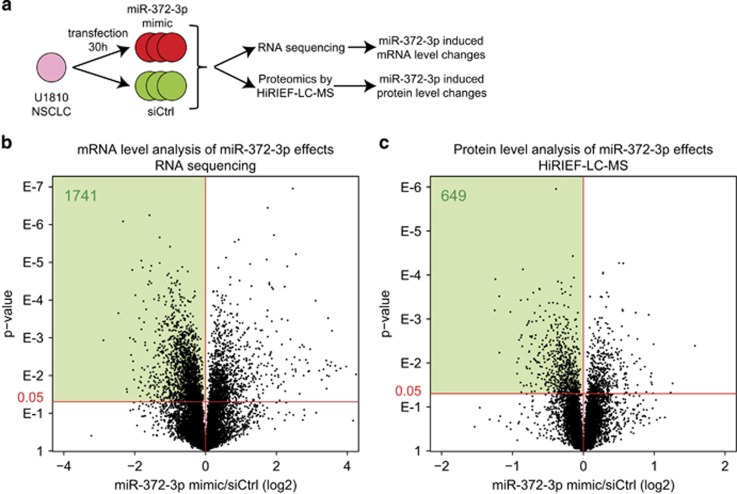
Global mRNA and protein expression analysis of miR-372-3p effects. (**a**) U1810 NSCLC cells were transfected in triplicates with miR-372-3p mimics or non-targeting siRNA control and analyzed by RNA sequencing and HiRIEF-LC-MS. (**b**) Volcano plot showing the mRNA-level analysis of miR-372-3p effects. Light green area indicates 1741 potential miR-372-3p targets (miR-372-3p/siCtrl log2<0, *P*-value<0.05). (**c**) Volcano plot showing the protein-level analysis of miR-372-3p effects. Light green area indicates 649 potential miR-372-3p targets (miR-372-3p/siCtrl log2<0, *P*-value<0.05).

Our next objective was to investigate the molecular consequences of AAGUGC-miRNA expression in relation to the detected phenotype. As miRNAs exert their cellular functions through targeting mRNAs, resulting in mRNA degradation, translation inhibition or both, we performed global mRNA- and protein-level analysis of cells treated with synthetic miR-372-3p mimics to identify potential AAGUGC-miRNA targets. Briefly, triplicate cultures of U1810 cells were transfected with miR-372-3p mimics or non-targeting siRNA and harvested 30 h after transfection (Figure 3a). The choice of time point was based on a previously published global analysis of miRNA effects at both protein and mRNA level.^11^ For the mRNA-level analysis of miR-372-3p effects, RNA sequencing was performed resulting in the identification and quantification of transcripts corresponding to 12 657 genes (Supplementary Table 2). The protein-level analysis was performed by high-resolution isoelectric focusing–liquid chromatography–mass spectrometry (HiRIEF-LC-MS) with relative quantification between samples using isobaric tags, and led to the identification and quantification of proteins corresponding to 9514 genes (Supplementary Table 3). Potential miR-372-3p target genes were defined as genes being downregulated at mRNA (1741) or protein (649) level in miR-372-3p mimic-treated cells compared with non-targeting siRNA treated cells (*P*-value cutoff of 0.05, Figures 3b and c).

**Figure 4:**
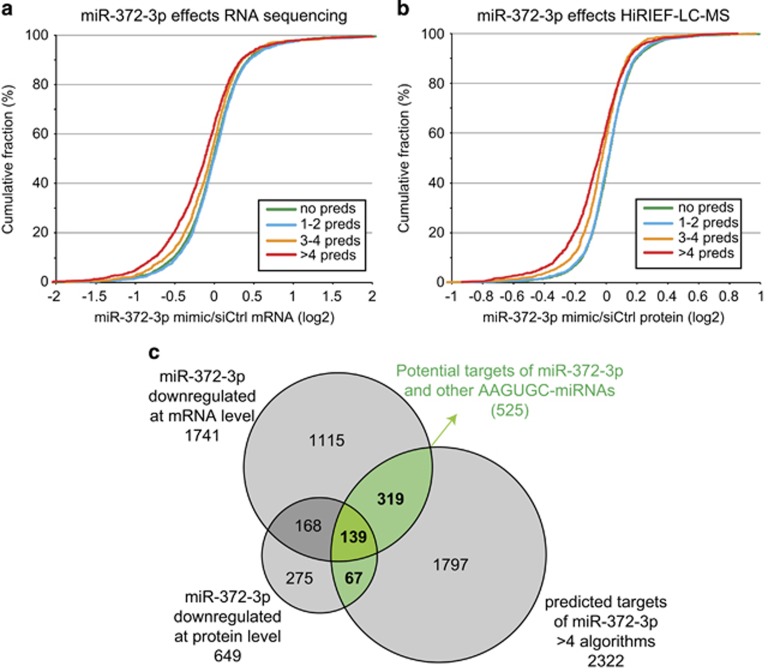
miR-372-3p target prediction and potential miR-372-3p targets. (**a**) Cumulative distribution of mRNA level fold changes upon treatment with miR-372-3p mimic, calculated for four different mRNA subsets: mRNAs predicted as targets of miR-372-3p by more than four different target prediction algorithms; three to four algorithms; one to two algorithms or no algorithms. (**b**) Cumulative distribution of protein-level fold changes upon treatment with miR-372-3p mimic, otherwise same as in (**a**). (**c**) Venn diagram showing the overlap between mRNA level analysis of miR-372-3p effect, protein-level analysis of miR-372-3p effects and miR-372-3p target prediction analysis (targets predicted by more than four algorithms).

mRNA targets of specific miRNAs can be predicted based on complementarity between the miRNA seed sequence and the mRNA 3'UTR sequence. To predict miR-372-3p targets, we used nine different miRNA target prediction algorithms through the miRWalk database.^12^ In total, 7508 different miR-372-3p targets were predicted, with the number of predictions per algorithm ranging from 451 (miRDB) to 6146 (PICTAR5) (Supplementary Figure 1a). To rank the predictions, we used the number of algorithms predicting the same target, ranging from targets predicted by a single algorithm (2154 mRNAs) to all nine (only 2 mRNAs) (Supplementary Figure 1b). The mRNAs and proteins quantified in response to miR-372-3p mimics were then stratified into four groups according to the number of algorithms predicting them as targets of miR-372-3p (no predictions, 1–2 predictions, 3–4 predictions and more than 4 predictions). The cumulative distribution of mRNA or protein fold changes upon treatment with miR-372-3p mimics across the four groups showed no general downregulation of mRNAs or proteins predicted by one to two algorithms, whereas a clear enrichment of downregulated mRNAs and proteins was evident in the group predicted by more than four algorithms (Figures 4a and b). Interestingly, the distribution of the number of algorithms predicting the same target mRNA was bimodal with a peak in targets predicted by five algorithms suggesting a threshold for higher algorithm consistency (Supplementary Figure 1b). For further analysis, we therefore focused on targets predicted by five or more algorithms.

Combining the global mRNA- and protein-level analysis of miR-372-3p targets with the target prediction analysis resulted in a list of 525 putative miR-372-3p targets that were downregulated at mRNA and/or protein level and also predicted as targets by more than four different algorithms (Figure 4c, Supplementary Table 4).

### miRNAs with the AAGUGC-oncomotif target a wide range of tumor suppressors with growth inhibiting functions

According to the miRNA database miRBase, the human genome contains a total of 28 different miRNAs with an AAGUGC motif in their seed sequence (Supplementary Figure 2). These 28 miRNAs are expressed from seven different loci, and in six of these loci, several different AAGUGC-miRNAs are clustered together. In two cases, these miRNA clusters are located within an intron of a so-called miRNA host gene (*MIR17HG* for miR-17~92 and *MCM7* for miR-106b~25), and in remaining cases, they are located in intergenic regions. Although it is likely that these different miRNAs have their own unique target mRNAs, our and others' data indicate that there is a common oncogenic phenotype for many of these miRNAs, suggesting that there is also a common set of target mRNAs. The performed analysis indicates that the AAGUGC-core motif present in the seed sequence of all these miRNAs is important for their oncogenic properties. For this reason, we will refer to this core motif as the oncomotif and to the group of 28 miRNAs as oncomotif-miRNAs.

**Figure 5:**
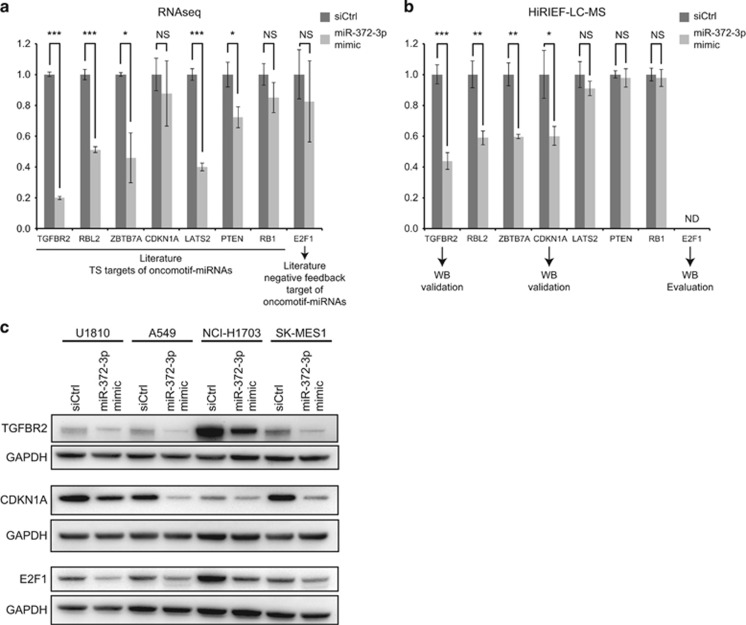
miR-372-3p reduces the expression of oncomotif-miRNA targets. (**a**) RNA sequencing results of several previously described and validated targets of different oncomotif-miRNAs. mRNA levels in miR-372-3p mimic-transfected cells are shown in relation to non-targeting siRNA ctrl-transfected cells. Indicated are also the results from a *t*-test analysis based on the RNA sequencing data (***P-value<0.001, **P-value<0.01, *P-value<0.05 and NS=non-significant). (**b**) Quantitative MS results of the same targets. Protein levels in miR-372-3p mimic-transfected cells are shown in relation to non-targeting siRNA ctrl-transfected cells. Indicated are also the results from a *t*-test analysis based on the MS analysis (*P*-values are indicated as in (**a**), NS=non-significant, ND=not determined). (**c**) Western blot analysis of miR-372-3p mimic effect on selected miR-372-3p targets in four different NSCLC cell lines. GAPDH was used as a loading control, and the figure shows representative blots from three replicate experiments.

To identify mRNA targets involved in the regulation of cell proliferation, we performed a literature search of previously described and validated targets of individual oncomotif-miRNAs. Our primary focus was directed towards TSs because inhibition of such targets would promote oncogenic growth. The literature search resulted in the identification of seven well-established TS targets previously described to be regulated by one or several different oncomotif-miRNAs (*TGFBR2,*^13^ *CDKN1A,*^14^ *LATS2,*^10^ *RBL2,*^15^ *ZBTB7A,*^16^ *PTEN*^17^ *and RB1*;^18^ for additional references and details, see Supplementary Table 5). All of these TS targets except *RB1* were among the 525 putative miR-372-3p targets here identified, suggesting that they are common oncomotif-miRNA targets (Figures 5a and b). Conversely, several oncomotif-miRNAs have also been shown to target E2F1, resulting in translational inhibition but no impact on mRNA stability, indicating that they may be involved in *negative* regulation of proliferation.^19^ Our experimental analysis of miRNA targets showed concordance between mRNA- and protein-level analysis in some cases (for example, TGFBR2, RBL2 and ZBTB7A), whereas in other cases, the regulation was only seen at one level (for example, LATS2 at mRNA level and CDKN1A at protein level). The overlap between the analyses was also not complete as illustrated by E2F1, which was not identified by mass spectrometry. To further evaluate our findings, we selected three oncomotif-miRNA targets (TGFBR2, CDKN1A and E2F1) for western blot validation in four different cell lines (U1810, A549, NCI-H1703 and SK-MES-1) using miR-372-3p mimics. The western blot data showed robust downregulation of the targets at the protein level in all cell lines (Figure 5c).

### Oncomotif-miRNA expression in lung adenocarcinoma is linked to E2F-driven cancer cell proliferation, TP53 mutation and MYC amplification

To investigate the clinical relevance of oncomotif-miRNAs in lung adenocarcinoma (LUAD), miRNA and mRNA expression data generated in The Cancer Genome Atlas (TCGA) project were downloaded from the UCSC Cancer Browser. The expression of oncomotif-miRNAs in 445 LUAD cases indicated dramatically different expression levels across the patient cohort, with hsa-mir-93 showing the highest expression followed by hsa-mir-17 and hsa-mir-106b (Supplementary Figure 3a). Even though hsa-mir-93 was the dominant oncomotif-miRNA in most tumors, sporadic cases of high expression was seen for most miRNAs as exemplified for the 10 tumors with the highest sum expression of oncomotif-miRNAs (Supplementary Figure 3b). The five miRNAs of the miR-302 family (hsa-mir-302 a–e) were not expressed in LUAD, which is in agreement with previous reports showing stem cell-specific expression of these miRNAs.^20^

**Figure 6:**
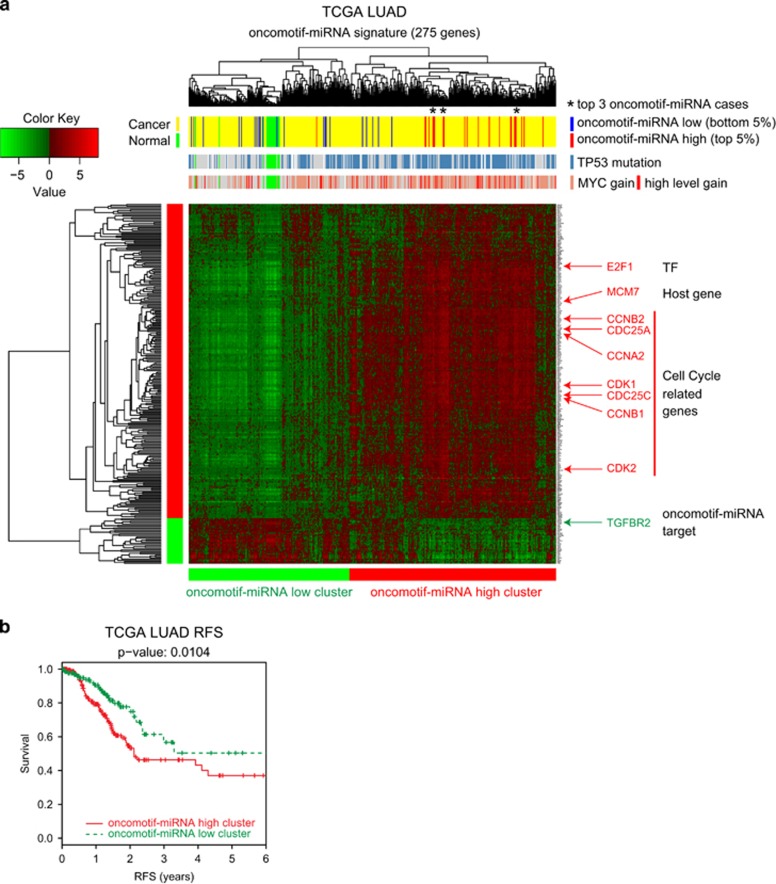
Cluster analysis of LUAD based on oncomotif-miRNA signature genes. (**a**) Heatmap showing the results of hierarchical clustering (patients horizontally and genes vertically) of 445 LUAD samples and 19 normal lung epithelium samples (TCGA LUAD) based on the mRNA expression of the 275 oncomotif-miRNA signature genes (see Supplementary Figure 4 for details). Heatmap colors represent mean centered log2 fpkm values. Inserts, from top to bottom, indicate; cancer (yellow), normal (green), oncomotif-miRNA high cancer (top 5%, red) and oncomotif-miRNA low cancer (bottom 5%, blue); TP53-mutated (blue) or TP53 wt (gray); MYC gain (light red), MYC high-level gain (red) or no gain (gray). Asterisks indicate the three cases with the highest oncomotif-miRNA expression. Indicated on the right are a few genes with increased expression (red, oncomotif-miRNA transcription factor, oncomotif-miRNA host gene and cell cycle related genes) or decreased expression (green, oncomotif-miRNA target) in the oncomotif-miRNA high compared with low clusters. (**b**) Kaplan–Meier plot of relapse-free survival (truncated at 6 years) for the oncomotif-miRNA high cluster patients (red, *n*=261) and oncomotif-miRNA low cluster patients (green, *n*=184).

Because of the overlapping target spectrum of oncomotif-miRNAs, we considered the sum of their expression for the continued analysis of the TCGA data. To investigate differences in signaling between tumors with high or low oncomotif-miRNA expression, we performed differential mRNA expression analysis between the top and bottom 5th percentiles of the 445 TCGA LUAD samples (Supplementary Figure 4a). Gene set enrichment analysis^21, 22^ indicated a strong association between oncomotif-miRNA expression and cell proliferation as both KEGG and Reactome cell cycle gene sets were enriched among the genes with higher expression in oncomotif-miRNA high tumors (Supplementary Figure 4b). Next, Ingenuity pathway analysis (www.ingenuity.com) was used to identify pathways or networks associated with oncomotif-miRNA expression by analyzing the top 275 genes separating the high and low expressing tumors (log2 abs(fold) >1, false discovery rate<1.0 E-05, Supplementary Figure 4c and Supplementary Table 6). The top scoring network in this analysis was centered on E2F1 (Supplementary Figure 4d), further supporting a role of oncomotif-miRNAs in regulation of tumor cell proliferation. Hierarchical clustering of the 445 LUAD samples and 19 normal lung samples based on the 275 oncomotif-miRNA signature genes resulted in a clear separation of the cohort into two main clusters (Figure 6a). One cluster (containing oncomotif-miRNA high tumors) was characterized by higher expression of a large number of genes driving cell proliferation (for example, *E2F1*, *CDK2*, *CDC25A*, *CCNA2*), while the other cluster (containing oncomotif-miRNA low tumors) also contained all normal lung samples. Importantly, tumors with high oncomotif-miRNA expression clustered together irrespective of the relative contribution of different miRNAs, as shown for the three cases with the highest oncomotif-miRNA expression (indicated with an asterisk) but different dominating miRNAs (C19MC cluster miRNAs, miR-371~373 cluster miRNAs and miR-93, respectively, Supplementary Figure 3b). When analyzing oncomotif-miRNA expression in relation to common genetic alterations in LUAD, the top two findings were significantly higher expression of oncomotif-miRNAs in tumors harboring *TP53* mutation and *MYC* amplifications (Supplementary Figures 5a and b). In addition, but with lower significance, oncomotif-miRNA expression was higher in tumors with mutated KEAP1 or RB1, and in tumors with TERT amplification. The association of the miRNA signature with clinical parameters was also investigated showing significantly lower relapse-free survival in patients with tumors belonging to the high oncomotif-miRNA cluster (Figure 6b). To test whether these results were independent of genetic alterations associated with oncomotif-miRNA expression, we performed univariate and multivariate survival analysis including also TP53, KEAP1 and RB1 mutation as well as MYC or TERT amplification. The only significant finding in this analysis was that patients with tumors belonging to the high oncomotif-miRNA cluster has shorter relapse-free survival (Supplementary Figures 5c and d). In summary, our analyses show that oncomotif-miRNA expression in LUAD is strongly associated with E2F-driven tumor cell proliferation, *TP53* mutation, *MYC* amplification and shorter relapse-free survival.

### Oncomotif-miRNAs are part of an oncogenic signaling network in LUAD

**Figure 7:**
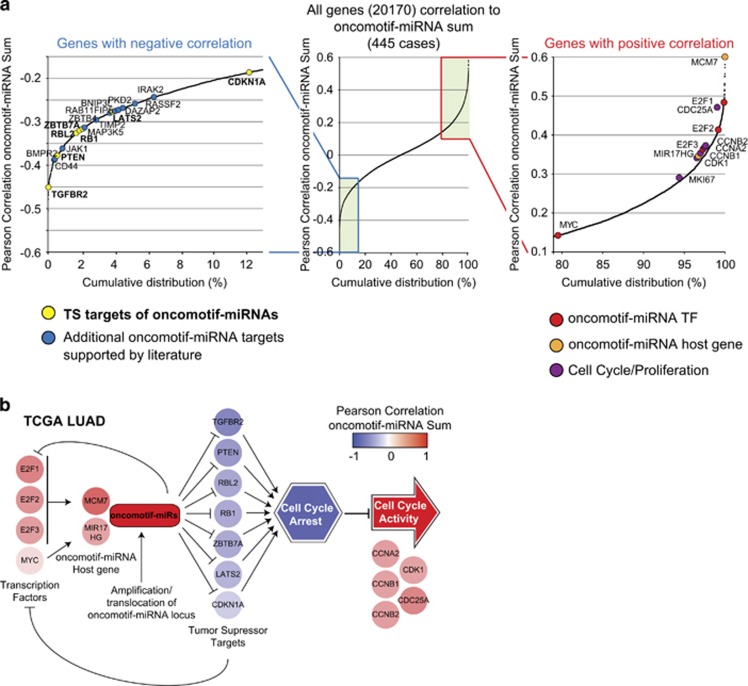
Oncomotif-miRNA signaling in LUAD. (**a**) Middle plot shows the cumulative distribution of correlations between oncomotif-miRNA expression and the mRNA expression of 20 170 genes in LUAD (TCGA LUAD). On the left side is a zoom in on the genes with a negative correlation to oncomotif-miRNA expression. Indicated in the plot are previously validated targets of different oncomotif-miRNAs (yellow are TS targets discussed here and blue are additional targets with literature support). On the right side is a zoom in on genes with a positive correlation to oncomotif-miRNA expression. Indicated in the plot are known oncomotif-miRNA transcription factors, oncomotif-miRNA host genes and cell cycle/proliferation-related genes. (**b**) Oncomotif-miRNA signaling network in LUAD. Individual genes are color-coded based on correlation to oncomotif-miRNA expression in TCGA LUAD. Activating E2F transcription factors (E2F1-3) and MYC drives expression of oncomotif-miRNAs through transactivation of known miRNA host genes *MCM7* (hsa-mir-93 and hsa-mir-106b) and *MIR17HG* (hsa-mir-17 and hsa-mir-20a). Sporadic high expression from additional oncomotif-miRNA loci through, e.g., amplification/translocation contributes to the total expression of oncomotif-miRNAs. Oncomotif-miRNA-dependent inhibition of common TS targets results in a relieved cell cycle arrest and progression through the cell cycle as indicated by cell cycle-related genes. Oncomotif-miRNA expression also form a self-propagating feed-forward loop as several of the TS targets are also inhibitors of the transcription factors responsible for oncomotif-miRNA expression. See also main text for extensive discussion.

As at least part of the impact of miRNAs on their targets is excerted through mRNA degradation, the correlation between miRNA expression and mRNA levels in clinical samples can provide information about targets that are clinically relevant. Out of the 525 potential targets defined here, 194 were found to inversely correlate with oncomotif-miRNA expression in the TCGA LUAD cohort (pearson correlation <−0.15, Supplementary Table 7). Importantly, all seven TS targets discussed here were found to inversely correlate with oncomotif-miRNA expression in LUAD (Figure 7a). Among these 194 candidate targets were also several other previously reported targets of individual oncomotif-miRNAs, supporting a common target spectrum. Furthermore, a positive correlation was seen between miRNA expression and known oncomotif-miRNA transcription factors (*E2F1-3* and *MYC*) and host genes (*MCM7* and *MIR17HG*). In addition, a strong positive correlation was observed between oncomotif-miRNA expression and cell cycle-related genes as well as the proliferation marker *MKI67*.

On the basis of our findings, we suggest that oncomotif-miRNAs are an integral part of a signaling network that regulates cell proliferation, and that overexpression of these miRNAs has the potential to promote oncogenic growth through the inhibition of a series of TS targets (Figure 7b).

### Pan-cancer oncomotif-miRNA analysis

To further investigate the oncomotif-miRNA expression pattern in the context of cancer, we used miRNA-profiling data generated in the TCGA project. Oncomotif-miRNA expression analysis in 32 different cancer types showed large differences in overall expression between cancer types (Supplementary Figure 6). The highest expression was seen in testicular germ cell cancer (TGCT, median number of oncomotif-miRNA reads per million miRNA reads mapped equaled 55 023) followed by acute myeloid leukemia (30 344) and thymoma (THYM, 26 629). Cancer types with low oncomotif-miRNA expression included pancreatic cancer (PAAD, 3493) and kidney cancer (KIRC, 3875 and KICH, 2587). The relative contribution of different oncomotif-miRNAs to the summed expression showed a general pattern with the highest expression of hsa-mir-93 followed by hsa-mir-17 in the majority of cancer types (Supplementary Figure 7a). A striking exception from this general pattern was noticed in TGCT where the miR-371~373 cluster was dominating together with the miR-302~367 and C19MC clusters. Also, THYM showed a specific oncomotif-miRNA expression pattern with relatively high expression of the C19MC cluster. Similar to the results in LUAD, sporadic high expression of oncomotif-miRNAs from the miR-371~373 and C19MC clusters was noticed in several other cancer types (Supplementary Figure 7b).

**Figure 8:**
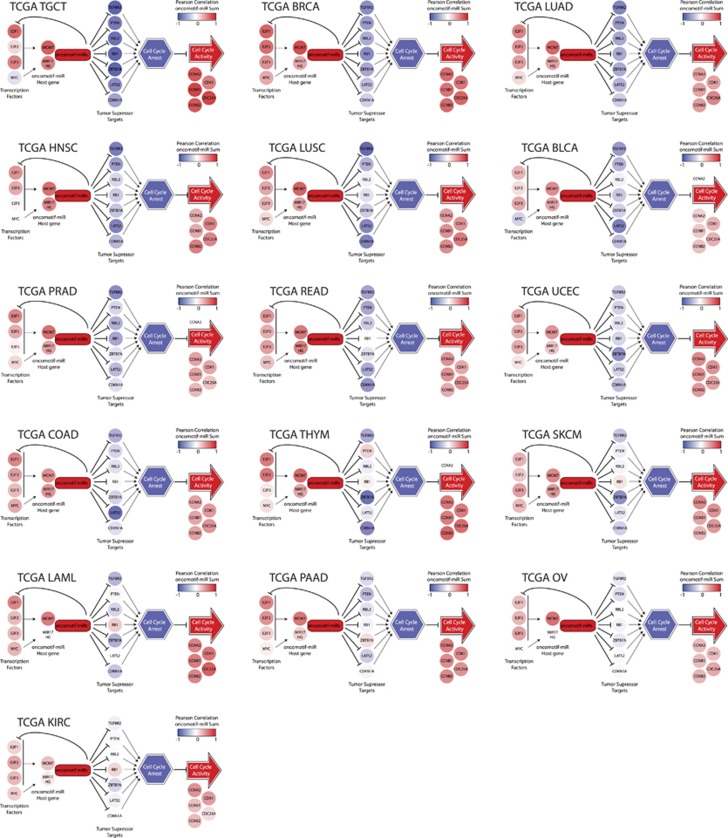
Oncomotif-miRNA signaling network in additional cancer types. Oncomotif-miRNA correlation networks in 16 cancer types based on miRNA and mRNA sequencing data (TCGA). Individual genes are color-coded based on the mRNA correlation to oncomotif-miRNA expression for each cancer type separately. Cancer types are ordered from left to right and top to bottom based on the strongest average negative correlation between oncomotif-miRNA expression and TS target genes. Cancer type abbreviations are: TGCT (testicular germ cell tumor), BRCA (breast carcinoma), LUAD (lung adenocarcinoma (AC)), HNSC (head and neck squamous cell carcinoma (SCC)), LUSC (lung SCC), BLCA (bladder urothelial carcinoma), PRAD (prostate AC), READ (rectum AC), UCEC (uterine corpus endometrial carcinoma), COAD (colon AC), THYM (thymoma), SKCM (skin cutaneous melanoma), LAML (acute myeloid leukemia), PAAD (pancreatic AC), OV (ovarian serous cystadenocarcinoma), KIRC (kidney renal clear cell carcinoma).

To evaluate a general role of oncomotif-miRNAs in oncogenic signaling, correlation networks for an additional 15 cancer types were produced through correlating the oncomotif-miRNA expression with mRNA expression levels from TCGA as was performed for LUAD (Figure 8). This analysis revealed that the correlations found in the LUAD analysis were present also for other cancer types. The strongest average negative correlation between oncomotif-miRNA expression and the seven TS targets was seen in TGCT, the cancer type with the highest expression of oncomotif-miRNAs. Conversely, the weakest negative correlation was seen in KIRC, the cancer type with the lowest expression of oncomotif-miRNAs of the cancer types analyzed. Despite these results, we did not see a direct connection between oncomotif-miRNA expression level and TS target correlation. As an example, the second and third strongest negative correlations were seen in breast (BRCA) and lung (LUAD) cancers, two cancer types with intermediate oncomotif-miRNA expression.

To further validate the results from the LUAD analysis, the breast cancer dataset (BRCA) was selected because it showed a generally high correlation between oncomotif-miRNAs and TS targets. Hierarchical clustering of 762 samples from BRCA patients and 87 normal samples based on the expression of the 275 oncomotif-miRNA signature genes defined in LUAD generated results that very closely mimicked the results in LUAD (Supplementary Figure 8a). Two main clusters were produced, one containing cancers with high oncomotif-miRNA expression showing higher expression of genes related to cell cycle activity and proliferation, and one cluster with low oncomotif-miRNA-expressing tumors and normal samples. Also, in BRCA, the expression of oncomotif-miRNAs was higher in tumors harboring *TP53* mutation and *MYC* copy number gain (Supplementary Figure 8b). In addition, although without reaching statistical significance, there was a trend for shorter relapse-free survival in BRCA patients in the oncomotif-miRNA-high cluster compared with the oncomotif-miRNA-low cluster, similarly to what was seen in lung cancer (Supplementary Figure 8c).

In summary, the analysis across multiple cancer types strongly supports the results of the LUAD analysis, indicating that oncomotif-miRNAs are in fact an integrated part of the oncogenic signaling network that drives cancer cell proliferation.

## Discussion

Our miRNA target analyses revealed broad oncomotif-miRNA targeting of *bona fide* TSs responsible for inhibition of cell proliferation. RB1 (pRb) and RBL2 (p130) are directly involved in inhibition of activating E2F transcription factors (E2F1-3) that transactivate genes crucial for G1/S progression. RB1 binds to and inhibits E2F1-3 directly, and RBL2 forms a repressor complex together with inhibitory E2F transcription factors (E2F4-5) resulting in repression of E2F1-3 target genes.^23^ CDKN1A (p21) is a cyclin-dependent kinase (CDK) inhibitor that binds to and inhibits the activity of cyclin/CDK complexes resulting in RB1-dependent inhibition of activating E2F transcription factors. TGFBR2 also has a negative impact on cell proliferation as TGFBR2 signaling results in the upregulation of several different CDK inhibitors including CDKN1A.^24^ PTEN is an inhibitor of the PI3K/AKT signaling pathway that promotes both proliferation and survival. The LATS2 TS is suggested to function through inhibition of MDM2 resulting in stabilization of the TS TP53 (p53),^25^ as well as directly inhibiting the activity of the cyclinE/CDK2 complex.^26^ ZBTB7A has been shown to be a TS in prostate cancer as ZBTB7A activity results in increased RB1 stability.^16^ Our analysis of oncomotif-miRNA targets included miRNA target prediction algorithms that predict targets based on complementarity between the seed sequence of the miRNA and the 3' UTR of mRNAs as well as evolutionary conservation in the 3' UTR sequence. It should be acknowledged that other parts of the miRNA, not considered by the prediction algorithms, also affect the binding to mRNA, resulting in both false-negative and false-positive predictions.^27^ Importantly, all of the TS targets here discussed have previously been described and validated as targets of different oncomotif-miRNAs. Our analysis here shows that expression of a single oncomotif-miRNA (miR-372-3p) is sufficient for targeting an entire panel of these tumor supressors. Our data also support previous studies showing that different oncomotif-miRNAs share common TS targets. Consequently, whenever two or more oncomotif-miRNAs are expressed in the same cell, they will jointly contribute to the silencing of those targets. In addition, our data demonstrate that high expression of oncomotif-miRNAs in cancer cells promotes cell cycle progression and increases proliferation.

In normal cells, expression of oncomotif-miRNAs through activating E2F transcription factors^14^ and MYC^19^ in response to, for example, growth factor signaling, could be beneficial to rapidly induce proliferation when needed. In analogy, it has been shown that a set of miRNAs that target immediate early genes responsible for cell cycle entry are rapidly downregulated in response to growth factor stimulation.^28^ Such signaling, however, needs to be carefully regulated not to become oncogenic and this is usually accomplished by efficient negative feedback loops. It has previously been suggested that several oncomotif-miRNAs themselves are part of such a negative feedback loop via targeting of activating E2F transcription factors.^19^ Even though we also detect E2F1 targeting, the overall analysis supports a powerful feed-forward loop through oncomotif-miRNA targeting of several TSs known to cause inhibition of E2F activity and G1/S progression. The oncomotif-miRNA-dependent increase in E2F activity would subsequently result in additional oncomotif-miRNA transcription, creating a self-propagating loop. This model is further supported by the fact that ectopic expression of oncomotif-miRNAs results in increased proliferation. Our data thus suggest that even if oncomotif-miRNAs target E2F transcription factors, the net effect when taking into account the TS targets of oncomotif-miRNAs is increased proliferation.

Oncogenic growth signaling, for example, through EGFR or KRAS, results in cyclinD/cdk4-dependent hyperphosphorylation of retinoblastoma protein (RB1), release of activating E2F transcription factors (E2F1-3) from RB1 inhibition and E2F-dependent transcription of genes involved in cell cycle progression. Our data suggest that E2F-dependent transcription of oncomotif-miRNAs (hsa-mir-93 and hsa-mir-106b)^14^ contributes to cell cycle progression by targeting TSs that would otherwise inhibit cell cycle entry. Likewise, oncogenic MYC signaling would result in inhibition of the same TSs through transcription of other oncomotif-miRNAs (hsa-mir-17 and hsa-mir-20a).^19^ The fact that hsa-mir-93 and hsa-mir-17 are the dominant oncomotif-miRNAs in most cancer cases suggests that oncogene-dependent transcription through MYC and E2Fs is an important contributor to oncomotif-miRNA expression in cancer, but the elucidation of specific mechanisms of oncomotif-miRNA expression in different cancer types warrants further investigation. Further, our analysis shows that in sporadic cases, oncomotif-miRNA expression from other genomic loci becomes dominant. Interestingly, three of the alternative loci (including miR-106a~363, miR-371~373 and miR-302~367 clusters) have been shown to be expressed specifically in embryonic stem cells during early embryogenesis^29^ and are important for proliferation.^30^ Sporadic re-expression of stem cell-specific miRNAs could potentially be a result of a genetic translocation, placing the miRNA locus under the control of an active promoter as previously shown for oncomotif-miRNAs of the C19MC and miR-371~373 clusters.^15, 31^ Additional mechanisms for altered regulation could include genetic imprinting, epigenetic regulation and antisense-mediated regulation as described for the miR-371~373 cluster.^32^ Importantly, cancer cases with high oncomotif-miRNA expression cluster together irrespective of the relative contribution of individual miRNAs, supporting an overlapping core set of target mRNAs and redundant oncogenic properties. This finding motivates the analytical approach used here, where different miRNAs with strong seed sequence homology are analyzed as a group. The grouped analysis also allows for identification of low frequency events that would otherwise have been difficult to find.

An important cellular defense mechanism against oncogene activation is through induction of permanent cell cycle arrest, a process termed oncogene-induced senescence.^33^ Both RB1 and TP53 are key regulators of the senescence program that includes and relies on the activation of several different CDK inhibitors including CDKN1A.^34^ It has previously been shown that several different oncomotif-miRNAs have the potential to rescue cells from oncogene-induced senescence by targeting CDKN1A^35^ or LATS2.^10, 35^ In addition, oncomotif-miRNA-dependent inhibition of TGFBR2 would result in further hampering of the senescence program by decreasing the level of additional CDK inhibitors activated by TGF-beta signaling.

Without a functional negative feedback regulatory system and with the oncogene-induced senescence system impaired, a final defense mechanism for the organism against oncogenic signaling is to activate the programmed cell death machinery. It is well known that increased E2F1 signaling as well as oncogenic MYC signaling can result in induction of apoptosis.^36, 37^ TP53 is generally described as the master regulator of apoptosis and it has been shown that the induction of oncogene-driven apoptosis is dependent, at least partially, on functional TP53. Consequently, a fully operational apoptosis system should have the capacity to efficiently protect against the oncogenic signaling described here. Unfortunately, the apoptosis system is commonly disabled in cancer, for example, through mutation of *TP53*, which would allow the oncogenic signaling to continue. We have shown here that the expression of oncomotif-miRNAs in both LUAD and breast cancer is significantly higher in tumors harboring *TP53* mutations, indicating that the oncogenic signaling including oncomotif-miRNAs is tolerated in cells with the apoptotic machinery inactivated.

Previous studies have described connections between all individual oncomotif-miRNA containing miRNA clusters and cancer as exemplified by miR-17~92 cluster in lymphoma,^38^ miR-106a~363 cluster in T-cell leukemia,^39^ miR-106b~25 cluster in gastric cancer,^14^ C19MC cluster in CNS-PNET,^40^ miR-371~373 cluster in testicular cancer^10^ and miR-302~367 cluster in germ cell tumors.^41^ Hence, there is a well-established association between individual oncomotif-miRNA containing miRNA clusters and cancer. Importantly, oncomotif-miRNAs are also members of a suggested oncogenic miRNA superfamily defined by a central GUGC core motif that was shown associated with targeting of TSs.^42^ Our analysis supports these studies, and further suggests that multiple different oncomotif-miRNAs will cooperate in driving proliferation through targeting of the same set of TSs. Our analysis also shows that the expression level of oncomotif-miRNAs varies dramatically across and within a large number of different cancer types, indicating that the importance of oncomotif-miRNA expression for cancer cell growth varies between cancer types and cancer cases. Furthermore, in lung adenocarcinoma (LUAD) and breast cancer (BRCA), our analysis indicates that high expression of oncomotif-miRNAs is connected with shorter relapse-free survival. Collectively, these findings suggest that oncomotif-miRNA expression analysis could contribute important information for prognostication and therapy prediction in certain cancer types and for certain drugs, as discussed below.

Our data demonstrate that expression of oncomotif-miRNAs not only results in increased proliferation, but also in increased sensitivity to EGFR inhibitors. A possible connection between these two findings could be that cells become addicted to the oncogenic signaling potentiated by oncomotif-miRNA expression. The addicted cells would then be more sensitive to treatments such as EGFR inhibitors that target upstream signaling. These findings have several potential clinical implications. The first and most obvious is that patients with certain cancer types driven by EGFR signaling, which also have a high expression of oncomotif-miRNAs, may benefit from treatment with therapies targeting EGFR. This hypothesis needs to be further investigated, and for such an investigation, the performance of oncomotif-miRNAs as biomarkers in this setting should also be assayed. Interestingly, the clinical relevance of circulating cell-free miRNAs as biomarkers in cancer is currently being investigated intensively.^43^ In fact, many different oncomotif-miRNAs have already been suggested as diagnostic and prognostic blood-based biomarkers for different cancer types. Whether circulating cell-free oncomotif-miRNAs can be used as predictive biomarkers for EGFR-TKI-based therapy remains to be tested.

Second, our analysis suggests that elevated oncomotif-miRNA expression in patients results in loss of the G1/S checkpoint leading to uncontrolled proliferation of cancer cells. Large ongoing efforts are made to investigate pharmacological strategies to restore the G1/S checkpoint to halt cancer cell proliferation, for example, through inhibition of CDK4/6 (palbociclib^44^). Clinical trials have demonstrated promising results of palbociclib in treatment of breast cancer^45, 46^ and active research is now focusing on identifying predictive markers for improved selection of patients for palbociclib treatment. Our study suggests that evaluation of tumor expression of oncomotif-miRNAs could contribute important information in this setting.

Finally, the discovery of oncomiRs and TS miRNAs during the last 15 years spawned the idea of targeting miRNAs for cancer treatment. In principle, such treatment would involve either inhibition of oncogenic miRNAs or administration/derepression of TS miRNAs as reviewed by Ling *et al.*^47^ Highly interesting in relation to oncomotif-miRNA expression in cancer, is the development of seed-targeting tiny locked nucleic acids.^48^ These 8-mer oligonucleotides can be designed to inhibit entire seed sequence families, which would make it possible to target all oncomotif-miRNAs simultaneously. Also, it was shown that, when systematically administered, unconjugated tiny locked nucleic acids showed uptake in breast tumors in mice, coinciding with long-term miRNA inhibition. In fact, it was subsequently shown that tiny locked nucleic acids targeting miRNAs with an AAAGUGCU-seed motif (for example, miR-17-5p and miR-93-5p) were able to reduce cancer cell proliferation and prolong survival in mouse models of medulloblastoma.^49^ It is tempting to speculate that targeting the entire oncomotif-miRNA group by tiny locked nucleic acids could be an efficient way of re-establishing the G1/S checkpoint in cancers with high expression of oncomotif-miRNAs. For such an approach, careful consideration would have to be taken regarding the effects on normal cells because these miRNAs have various functions also in non-cancer cells.

In conclusion, our data suggest that oncomotif-miRNAs are an integral part of an oncogenic signaling network, and that oncomotif-miRNAs form a feed-forward loop promoting cell proliferation. In cancer, especially when pushed by MYC amplification and without the inhibitory effects of a functional TP53 system, this feed-forward loop becomes a self-propagating, continuous oncogenic driver of uncontrolled cell growth. In addition, we show that knowledge of oncomotif-miRNA expression in cancer patients may contribute valuable information in terms of prognostication, and even more importantly for selection of therapy.

## Materials and methods

### Cell lines and treatments

NSCLC cell lines U1810,^50^ A549 (ATCC, Rockville, MD, USA, CCL-185), NCI-H1703 (ATCC, CRL-5889) and SK-MES-1 cells (ATCC, HTB-58) were cultured in RPMI-1640 AQ media (Sigma-Aldrich, St Louis, MO, USA, R2405). Testicular germ cell cancer cell line 833KE (Sigma-Aldrich, 06072611) was cultured in Dulbecco's modified Eagle's medium (Sigma-Aldrich D0819). All media were supplemented with 10% fetal bovine serum (Sigma-Aldrich F6178) and 1% penicillin/streptomycin (Sigma-Aldrich P4333) at 37 °C and 5% CO_2_. All cell lines were tested and found *Mycoplasma*-free using MycoAlert Mycoplasma detection kit (Lonza, Walkersville, MD, USA, Cat. No. LT07-218). Gefitinib was purchased from Selleckchem (Houston, TX, USA, Cat. No. S1025).

For miR-Lib virus production, EcoPack II cells were cultured in Dulbecco's modified Eagle's medium (41966, Invitrogen, Grand Island, NY, USA) supplemented with 10% fetal calf serum and antibiotics (complete medium). Retrovirus was made by polyethyleneimine transfection of EcoPack II cells. The pMSCV–miR constructs were made as described previously.^10^ All miRNA transfection and virus collection steps were carried out on a Hamilton ML STAR (Hamilton Bonaduz, Bonaduz, Switzerland). Protocols were developed at the Netherlands Cancer Institute using Hamilton STAR Software 3.2. The methods were completely automated.

For proteomics, RNA sequencing and western blot experiments, cells were transfected with 10 nM Syn-hsa-miR-372-3p miScript miR-mimic (Qiagen, Hilden, Germany, Cat. No. MSY0000724), 10 nM AllStar neg. control siRNA (siCtrl, Qiagen, Cat. No. 1027281, thoroughly tested and validated nonsilencing siRNA) or 10 nM AllStars hs cell death siRNA (Qiagen, Cat. No. 1027298, positive cell death phenotype transfection control). Transfection reagent used in this study was Lipofectamine RNAiMAX (Thermo Fisher Scientific, Waltham, MA, USA, 13778-150). Thirty-two hours (RNAseq and proteomics) or 48 h (western blot) after transfection, cultures in biological triplicates for each condition (miR-372-3p mimic and control siRNA) were prepared and processed as described below for extraction of proteins and RNA.

For phenotype validation (flow cytometry and clonogenic assay) of miR-372 effects, stable hsa-mir-372-expressing cell lines and control cell lines were generated as follows. GFP-containing plasmids were purchased from SBI (System Biosciences, Mountain View, CA, USA, pre-miR-372 expression plasmid product number: PMIRH372PA-1; control plasmid product number: PMIRH000VA-1). The plasmids were transformed into *E.coli* separately and plasmid DNA was extracted using GeneJET plasmid midiprep kit (Thermo Fisher Scientific, K0482) according to product protocol. Lentiviruses were produced by transfecting plasmids into the virus packing cell line HEK293T together with Gag-pol/Rev/Envelope VsV-g pantropic plasmid (a gift from Professor Thomas Helleday Lab at Karolinska Institutet). Viruses were harvested every 12 h by collecting virus-containing culture media and used to infect target cell lines at the time of harvest. In total, target cell lines were infected three times. Virus-infected cells were sorted with BD Influx Cell Sorter (BD Biosciences, San Diego, CA, USA) using 140 micron nozzle tip. High GFP expression cell population (more than 1000 times GFP expression than basal cells) were sorted, collected and expanded for further experiments. Validation of GFP expression was performed by seeding 10 000 cells from each cell line in 12-well plates; GFP photos were taken with Bio-Rad ZOE Fluorescent Cell Imager (Bio-Rad, Hercules, CA, USA) to analyze expression of the constructs.

### miRNA dropout screen

Polyclonal pool of U1810 cells stably carrying the murine ecotropic receptor were generated to allow infections with ecotropic retroviral supernatants as described previously.^51^ U1810 cells were individually transduced with approximately 450 miRNA vectors (miR-Vecs) from a miRNA expression library (miR-Lib; for details see Voorhoeve *et al.*^10^ and Huang *et al.*^52^) using retroviral infection and drug selection (Blasticidin 5 ug/ml) to obtain resistant growing cells, each containing a unique integrated miR-Vec. Around 80% of vectors yielded stable clones. Cells were then pooled and plated at 1 000 000 cells per 10 cm dish for the three different conditions (Ctrl0, Ctrl30 and Gef30) in biological triplicates. Twenty-four hours after seeding, triplicate cultures were harvested for use as the baseline reference (Ctrl0). Ctrl30 and Gef30 cultures were cultured in separate biological triplicates in the absence or presence of 10 uM gefitinib and the medium was refreshed twice per week for 30 days and cells were split when needed to avoid confluency. After harvesting of cells, genomic DNA was isolated using QiaAmp kit (Qiagen) according to the manufacturer's protocol. miR-Vec inserts were recovered from 250 ng of genomic DNA by Phusion PCR amplification (Thermo Fisher Scientific) according to the manufacturer's protocol using primers specific for the pMSCV vector. Indexes and adaptors for deep sequencing (Illumina) were incorporated into PCR primers in two consecutive PCR reactions. PCR products were retrieved by electrophoresis followed by cutting out bands and purification using QIAEX II gel extraction kit (Qiagen). Deep sequencing was performed using the Illumina platform (Illumina, San Diego, CA, USA) at the NKI Central genomics facility. Sequencing indexes and miR-Vec sequences were segregated and deconvoluted from each sequencing read. Samples were normalized to total reads. We arbitrarily considered only miR-Vecs that had been sequenced at least 300 times in all three biological replicates of one condition, resulting in 140 unique miR-Vec miRNAs remaining for the enrichment/depletion analysis. *P*-values for enrichment/depletion analysis were calculated using two-sided *t*-test without correction for multiple testing.

### Protein extraction, digestion and Isobaric labeling

For extraction of proteins, pellets (biological triplicates) containing five million cells were lysed with sodium deoxycholate buffer (5% sodium deoxycholate, 1 mM dithiothreitol (Sigma-Aldrich, Product No. 43819), 25 mM HEPES), vortexed and kept on ice for 10 min, and then boiled in 95 °C for 10 min followed by sonication (2 cycles of 30 s, 80% energy). Vials were centrifuged at 11 000 r.p.m. for 15 min. Supernatants containing proteins were collected into new vials. Protein concentration was determined by Bio-Rad Quick Start Bradford protein assay.

Protein extracts were digested using trypsin (Thermo Fisher Scientific, 90058) using a filter-aided sample preparation protocol.^53^ The peptides generated from each sample were subsequently individually labeled with tandem mass tag (TMT)-10plex isobaric label reagents (Thermo Fisher Scientific, Cat. No. 90110). After pooling, the labeled peptides were cleaned by strong cation exchange solid-phase extraction (Phenomenex Strata-X-C, Phenomenex, Torrance, CA, USA) and aliquoted to 200 μg peptides mixture for isoelectric focusing (IEF).

### Peptide-level isoelectric focusing (HiRIEF)

Each labeled peptide pool (200 μg) was dissolved in 250 μl of rehydration solution (8 M urea, 1% pharmalyte for pH range 3–10 from GE Healthcare, Little Chalfont, UK), which was then used to re-swell an immobilized pH gradient gel-strip (GE Healthcare) pH 3–10. For the IEF in the 3.7–4.9 pH range, 200 μg of labeled peptide pool were dissolved in 150 μl of 8 M urea, and this solution was used to rehydrate a sample gel bridge (pH 3.7) overnight. The 3.7–4.9 immobilized pH gradient strip was rehydrated in 8 M urea, 1% pharmalyte for pH range 2.5–5 (GE Healthcare). All IEFs were run on an Ettan IPGphor (GE Healthcare) until at least 150 kVh for the 3–10 range and until at least 250 kVh for the 3.7–4.9 range (~1 day running time in either case). After focusing was complete, a well-former with 72 wells was applied onto each strip, and liquid-handling robotics (GE Healthcare prototype) added MilliQ water and, after 3 × 30 min incubation/transfer cycles, transferred the 72 fractions into a microtiter plate (96 wells, V-bottom, Corning, Lowell, MA, USA, Cat. No. 3894), which was then dried in a SpeedVac.

### LC-MS/MS analysis

For each LC-MS run of a HiRIEF fraction, the auto sampler (Ultimate 3000 RSLC system, Thermo Fisher Scientific) dispensed 15 μl of mobile phase A (95% water, 5% dimethylsulfoxide, 0.1% formic acid) into the corresponding well of the microtiter plate, mixed by aspirating/dispensing 10 μl ten times, and finally injected 7 μl into a C18 guard desalting column (Acclaim pepmap 100, 75 μm × 2cm, nanoViper, Thermo Fisher Scientific). After 5 min of flow at 5 μl/min with the loading pump, the 10-port valve switched to analysis mode in which the NC pump provided a flow of 250 nl/min through the guard column. The curved gradient (curve 6 in the Chromeleon software) then proceeded from 3% mobile phase B (90% acetonitrile, 5% dimethylsulfoxide, 5% water, 0.1% formic acid) to 45% B in 50 min followed by wash at 99% B and re-equilibration. Total LC-MS run time was 74 min. We used a nano EASY-Spray column (pepmap RSLC, C18, 2 μm bead size, 100 Å, 75 μm internal diameter, 50 cm long, Thermo Fisher Scientific) on the nano electrospray ionization EASY-Spray source (Thermo Fisher Scientific) at 60 °C. Online LC-MS was performed using a hybrid Q-Exactive mass spectrometer (Thermo Fisher Scientific). FTMS master scans with 70 000 resolution (and mass range 300–1700 m/z) were followed by data-dependent MS/MS (35 000 resolution) on the top five ions using higher energy collision dissociation at 30% normalized collision energy. Precursors were isolated with a 2 m/z window. Automatic gain control targets were 1e6 for MS1 and 1e5 for MS2. Maximum injection times were 100 ms for MS1 and 150 ms for MS2. The entire duty cycle lasted ~1.5 s. Dynamic exclusion was used with 60 s duration. Precursors with unassigned charge state or charge state 1 were excluded. An underfill ratio of 1% was used.

### Proteomics database search pipeline

Raw MS/MS files were converted to mzML format using msconvert from the ProteoWizard tool suite.^54^ Spectra were then searched using MSGF+^55^ (v10072) and Percolator^56^ (v2.08), where eight subsequent search results were grouped for Percolator target/decoy analysis. The reference database used was the human protein subset of ENSEMBL 79. MSGF+ settings included precursor mass tolerance of 10 p.p.m., fully tryptic peptides, maximum peptide length of 50 amino acids and a maximum charge of 6. Fixed modifications were TMT-10plex on lysines and N-termini, and carbamidomethylation on cysteine residues, a variable modification was used for oxidation on methionine residues. Quantification of TMT-10plex reporter ions was carried out using OpenMS project's IsobaricAnalyzer^57^ (v2.0). Peptide spectrum matches found at 1% false discovery rate were used to infer gene identities, which were quantified using the medians of peptide spectrum matches quantification ratios. *P*-values for protein-level regulation in response to miR-372-3p mimics were calculated using two-sided *t*-test without correction for multiple testing. The mass spectrometry proteomics data have been deposited to the ProteomeXchange Consortium via the PRIDE partner repository with the dataset identifier PXD004163.

### RNA sequencing and mapping

Total RNA was prepared in biological triplicates from U1810 cells (transfected and cultured as described above) using RNeasy Plus Mini Kit (Qiagen, 74134) according to product protocol. Total RNA concentration was measured using Qubit fluorometer (Invitrogen). RNA quality was assessed using LabChip GX-Caliper with HT 5 K/RNA LabChip, Ver2 (Perkin Elmer, Waltham, MA, USA) according to the manufacturer's instructions, and all samples showed high quality (RIN values>9). RNA libraries for sequencing were prepared using TruSeq Stranded mRNA Sample prep kit with 96 dual indexes (Illumina) according to the manufacturers instructions with the following changes. The protocols were automated using an Agilent NGS workstation (Agilent, Santa Clara, CA, USA) using purification steps as previously described.^58, 59^ Clonal clusters were generated using cBot (Illumina) and sequencing was performed on HiSeq2500 (Illumina) with a read length of 2 × 125 bases, generating an average number of reads per sample of 20.7 million read pairs per sample (ranging from 16.8 to 28 million read pairs). Mapping of the raw reads was performed using Tophat/2.0.4 to the Human genome assembly build GRCh37. Raw read counts were calculated with HTSeq (http://www-huber.embl.de/users/anders/HTSeq/) v0.5.1 on bam files with duplicates included. Differential expression analysis was performed on total read normalized values. Analysis was performed on all genes with a gene biotype annotated in ENSEMBL as protein coding. Genes with missing reads in any of the samples were removed from the analysis. *P*-values for mRNA level regulation in response to miR-372-3p mimics were calculated using two-sided *t*-test without correction for multiple testing. The RNA sequencing data have been deposited to the Gene Expression Omnibus (GEO) with the accession number GSE81417.

### Reverse transcription–quantitative PCR

miRNAs were extracted from 50 000 cells in biological triplicates from each cell line (A549, U1810, NCI-H1703, SK-MES1 and 833KE) and type (parental cells (PC), GFP-control cells (CC) and hsa-mir-372-transduced cells (MC)) using *mir*Vana miRNA isolation kit with phenol (Thermo Fisher Scientific, AM1560). miR-372-3p or the reference small RNA RNU48 were reverse-transcribed using Bio-Rad MyCycler thermal cycler with TaqMan MicroRNA Reverse Transcription Kit (Thermo Fisher Scientific, 4366596) according to product protocol, and miR-372-3p or RNU48 RT primer from TaqMan MicroRNA Assay (Life Technolgies, 4427975. RNU48 assay id: 001006 and miR-372 assay id: 000560). NTC (Non-Template Control) and NRT (Non-Reverse Transcriptase control) were included in the cDNA reverse transcription reaction. One microliter out of 15 μl cDNA product was used in each qPCR reactions, pipetted into Hard-Shell PCR plates 96-well thin wall (Bio-Rad, HSP9631) with TaqMan Universal Master Mix II no UNG (Thermo Fisher Scientific, 4440048) and analyzed using Bio-Rad CFX96 C1000 Touch Real-Time PCR detection systems according to the protocol. Relative fluorescence unit and quantitation cycle (abbreviated as C_q_) were used to calculate ΔΔC_q_ as follows: ΔC_q_ for each biological replicate was calculated by normalizing miR-372-3p C_q_ to RNU48 C_q_ (ΔC_q_=C_q(miR-372)_ - C_q(RNU48)_). Log2-transformed ΔC_q_ values were used to calculate triplicate average and standard deviation. Finally, ΔΔC_q_ was calculated by normalizing the mean of ΔC_q_ expression to non-targeting control (NTC), using the equation ΔΔC_q_=mean of ΔC_q_ expression/NTC mean ΔC_q_ expression. The whole calculation was performed with Bio-Rad CFX Manager 3.1 (Scan mode: SYBR/FAM only; Analysis mode: baseline substracted curve fit). Finally, as miR-372-3p expression was undetectable in all parental cells and GFP-control cells, miR-372-3p expression in the four NSCLC cell lines was reported in relation to the endogenous expression of miR-372-3p in 833KE cells.

### Cell cycle analysis

From each cell line (A549, U1810, NCI-H1703 and SK-MES1) and type (parental cells (PC), GFP-control cells (CC) and hsa-mir-372-transduced cells (MC)), 50 000 cells in biological triplicates were trypsinized, collected into fluorescence-activated cell sorting tubes, washed with 2 ml phosphate-buffered saline and centrifuged down with 1200 r.p.m. for 5 min. Supernatants were removed, leaving about 100 μl of phosphate-buffered saline, and the pellets were resuspended by flicking the tubes. Cells were fixed by incubating 1 h at 4 °C with 1 ml cold 70% ethanol and stained with Citrate buffer (0.05 M Na_2_HPO_4_, 25 mM sodium citrate dehydrate, 0.1% Triton X-100), 200 μl propidium iodide (Sigma-Aldrich P4863, 100 μg/ml in H_2_O) and 100 ul RNase (Sigma-Aldrich R6513, 100 μg/ml in phosphate-buffered saline) for 15 min at room temperature. The total amount of DNA was detected with Novocyte Flow Cytometer (ACEA Biosciences, San Diego, CA, USA). Cell cycle phases were automatically determined by NovoExpress software using the cell cycle analyze function and the read out contains G0/G1, S, G2/M phase cells percentile. Proliferation index, defined as the ratio between S, G2/M cells and G0/G1 cells, was calculated as previously described.^60, 61^ *P*-values were calculated using two-sided *t*-test without correction for multiple testing.

### Clonogenic assay

Clonogenic assay was performed as previously described.^62^ Briefly, 1000 cells from each cell line (A549, U1810 and NCI-H1703) and type (parental cells (PC), GFP-control cells (CC) and hsa-mir-372-transduced cells (MC)) were seeded in Corning Costar 6-well flat bottom cell culture plates (Sigma-Aldrich, CLS3516). Cells were cultured for 12 days in RPMI-1640 AQ media with or without 10 μM gefitinib, new media were replaced every 3 days. Each condition was analyzed in biological triplicates. Plates were rinsed with phosphate-buffered saline and incubated with 2 ml of 6% glutaraldehyde and 0.5% crystal violet for at least 30 min in room temperature. All the staining solutions were removed and plates were rinsed with water. Plates were then left to dry at room temperature in normal air before pictures were taken. Representative pictures from one of the replicates of each analysis is shown.

### Western blot

For western blotting, 0.8 million cells were transfected as described above and harvested by trypsinization 48 h after transfection. Cells were lysed with CHAPS buffer (1% CHAPS, 0.1% Triton X-100, 20 mM HEPES pH 7.5, 150 mM NaCl) and protein concentration was determined by Bio-Rad Quick Start Bradford protein assay. Samples were diluted in 4 × Laemmli Sample buffer (Bio-Rad, 1610747) with 1:10 β-mercaptoethanol. Samples were boiled at 95 °C for 5 min and run on a 10% TGX-gel (Bio-Rad, 4561036) at 150 V for 1 h. Proteins were transferred onto a mini nitrocellose membrane (Bio-Rad, 1704158) using Bio-Rad's Trans-Blot Turbo system at 1.3 A for 7 min. Membranes were blocked with 5% milk in TBS Tween buffer (28360, Thermo Fisher Scientific), for 1 h at room temperature and incubated with primary antibodies against TGFBR2 (Santa Cruz Biotechnology, Dallas, TX, USA, sc-33929), CDKN1A (Cell Signaling, Boston, MA, USA, 2947), E2F1 (Cell Signaling, 3742) and GAPDH (Sigma-Aldrich, G8795) overnight at 4 °C. Membranes were washed 3 × 10 min with TBS, incubated with secondary antibody (goat anti-mouse or goat anti-rabbit) conjugated with HRP at room temperature for 1 h. Membranes were developed with ECL (GE Healthcare, RPN2108) according to the manufacturer's instructions, and images were generated using ChemiDoc MP system (Bio-Rad). All western blot experiments were performed in biological triplicates. Representative images from one replicate experiment is shown.

### TCGA data analysis

Data from TCGA were accessed via the UCSC Cancer Browser. miRNA expression data were retrieved for all 32 cancer types and gene expression data for 16 cancer types. Copy number data, gene-level mutation data as well as clinical information were further retrieved for LUAD and breast invasive carcinoma (BRCA).

Differential gene expression analysis was conducted to identify genes differentially expressed between tumors with high or low oncomotif-miRNA expression. A two-sided *t-*test was performed between the top and bottom 5 percentiles of the TCGA LUAD samples (*n*=445). *P*-values were adjusted for multiple testing using the false discovery rate with the procedure outlined by Benjamini and Hochberg.^63^ All analyses were performed in R (version 3.2.2) using the *t.test* and *p.adjust* functions from the R *stats* package. The oncomotif-miRNA signature was defined by selecting all genes with a false discovery rate<1.0 E-05 and an absolute log2 fold-change >1. The signature comprises 275 genes, including 240 upregulated and 35 downregulated genes.

Unsupervised cluster analysis of the LUAD and BRCA gene expression profiles was performed using the 275 genes of the oncomotif-miRNA signature. All patients and genes were clustered using hierarchical clustering with complete linkage and Spearman's correlation. Heatmaps were produced using the *heatmap.2* function from the gplots package in R.

Differences in oncomotif-miRNA expression in the LUAD and BRCA data sets between tumors harboring TP53 mutations and MYC amplifications were examined using the Wilcoxon rank-sum test from the R *stats* package.

Univariate and multivariate survival analyses, including Cox proportional hazard regression and Kaplan–Meier survival curves, were carried out in R using the stats package. Patients were split into two groups using the two main clusters defined by the hierarchical clustering based on the oncomotif-miRNA signature. Cluster membership, mutation status and copy number status were all modeled as categorical variables. For the Kaplan–Meier analyses, the differences in survival time between two groups were assessed using the *P*-value of the survdiff function. A significance level of 5% was considered as statistically significant.

### miRNA target prediction, gene set enrichment analysis and Ingenuity pathway analysis

All miRNA target predictions were performed on 5 November 2014 using the miRWalk^12^ portal and standard settings (3′UTR sequences, minimum seed length:7, *P*-value:0.05). At that time, the available algorithms in addition to the miRWalk algorithm were: DIANAmT (version 3.0); miRanda (August 2010 release); miRDB (April 2009 release); PICTAR5 (March 2007 release); PITA (August 2008 release); RNA22 (May 2008 release); RNAhybrid (version 2.1) and TargetScan (version 5.1). For gene set enrichment analysis,^21, 22^ a ranked list of log2 ratios between the oncomotif-miRNA high and low groups (see results section) was loaded into the gene set enrichment analysis software. For gene set enrichment analysis, the C2:canonical pathways gene sets were used, and the analysis was performed using standard settings. Ingenuity pathway analysis (www.ingenuity.com) was performed by loading the 275 genes in the oncomotif-miRNA signature gene list with corresponding log2 ratios between the oncomotif-miRNA high and low groups into the ingenuity software. As background, the Ingenuity standard background gene list was used, and the analysis was performed using standard settings.
