# Supplementary material for: microRNAs with AAGUGC seed motif constitute an integral part of an oncogenic signaling network
Source: Oncogene. 2016 Aug 1;36(6):731–45. doi: 10.1038/onc.2016.242 (PMC5311252; doi:10.1038/onc.2016.242)
Supplement: Supplementary Information [file onc2016242x2.pdf]

Supplementary Figures related to:

microRNAs with an AAGUGC seed motif constitute an integral part of a signaling network driving NSCLC cell proliferation

Authors:

*Zhou Y[1], Frings O[1], Branca RM[1], Boekel J[1,2], le Sage C[3], Fredlund E[1], Agami R[3] and Orre LM[1]**

Affiliations:

[1]Department of Oncology-Pathology, Science for Life Laboratory, Karolinska Institutet, Stockholm, Sweden.
[2] Bioinformatics Infrastructure for Life Sciences (BILS), Stockholm, Sweden.
[3]Division of Biological Stress Response, the Netherlands Cancer Institute, Amsterdam, the Netherlands.
**Correspondence should be addressed to L.M.O. (lukas.orre@ki.se).*

Supplementary Figures

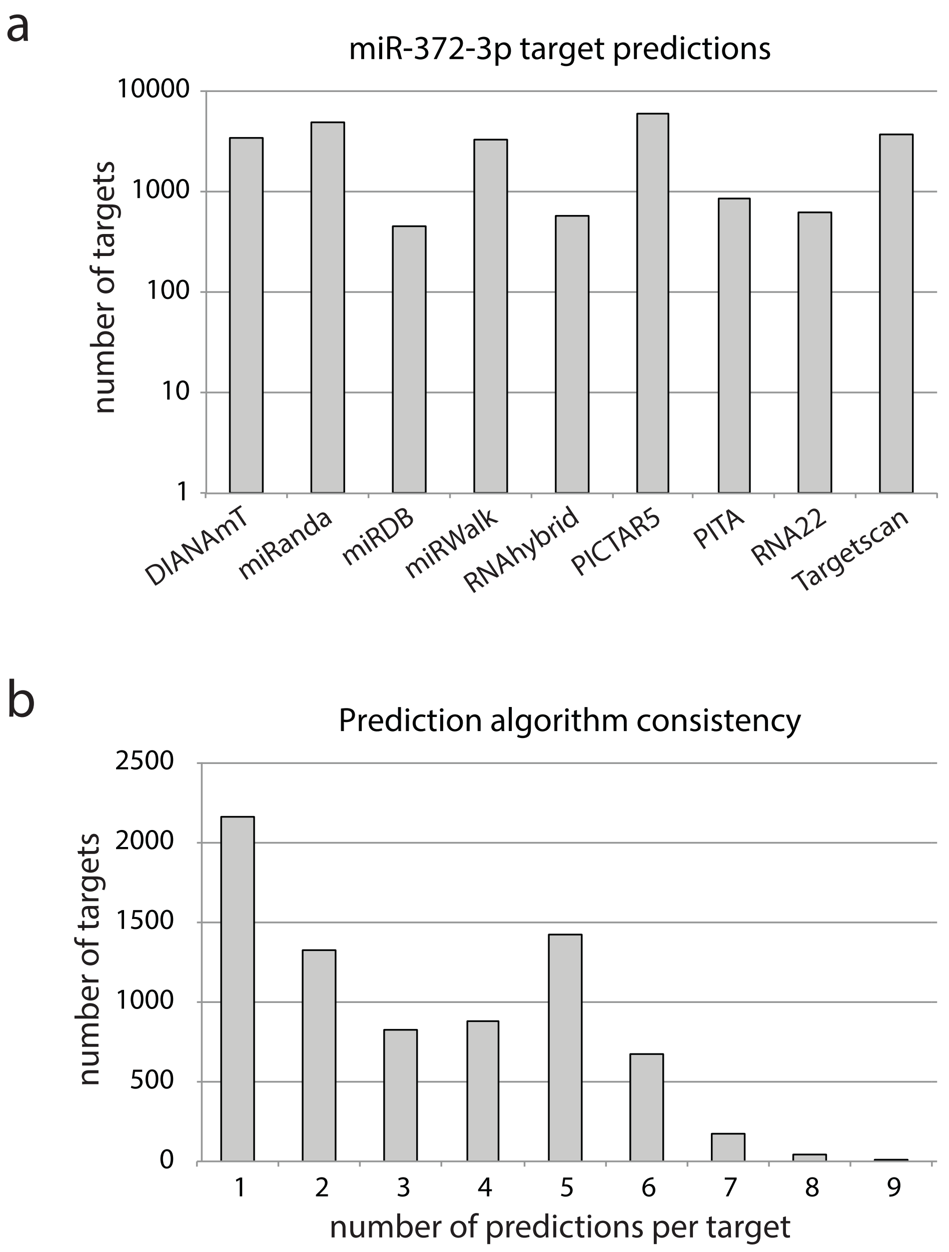

Supplementary Figure 1. miR-372-3p target prediction algorithm overview
a. Nine different miRNA target prediction algorithms were used for prediction of miR-372-3p targets. A total of 7,508 different targets were predicted, with the number of predictions per algorithm ranging from 451 (miRDB) to 6,146 (PICTAR5).
b. An evaluation of the prediction algorithm consistency showing the number of algorithms predicting the same target, ranging from targets predicted by a single algorithm (2,154 mRNAs) to all nine (only 2 mRNAs). In addition, a local maximum is evident for targets predicted by 5 different target prediction algorithms.

28 miRNAs in the human genome with AAGUGC-motif in seed sequence

microRNA ID	Mature miRNA	Mature Sequence	Chromosome	Genomic context	miRNA cluster
hsa-mir-17	miR-17-5p	**CAAAGUGC**UUACAGUGCAGGUAG	Chr 13	MIR17HG intron	miR-17~92 cluster
hsa-mir-20a	miR-20a-5p	**UAAAGUGC**UUAUAGUGCAGGUAG			
hsa-mir-106a	miR-106a-5p	**AAAAGUGC**UUACAGUGCAGGUAG	Chr X	Intergenic	miR-106a~363 cluster
hsa-mir-20b	miR-20b-5p	**CAAAGUGC**UCAUAGUGCAGGUAG			
hsa-mir-106b	miR-106b-5p	**UAAAGUGC**UGACAGUGCAGAU	Chr 7	MCM7 intron	miR-106b~25 cluster
hsa-mir-93	miR-93-5p	**CAAAGUGC**UGUUCGUGCAGGUAG			
hsa-mir-302a	miR-302a-3p	**UAAGUGCU**UCCAUGUUUUGGUGA	Chr 4	Intergenic	miR-302~367 cluster
hsa-mir-302b	miR-302b-3p	**UAAGUGCU**UCCAUGUUUUAGUAG			
hsa-mir-302c	miR-302c-3p	**UAAGUGCU**UCCAUGUUUCAGUGG			
hsa-mir-302d	miR-302d-3p	**UAAGUGCU**UCCAUGUUUGAGUGU			
hsa-mir-302e	miR-302e	**UAAGUGCU**UCCAUGCUU	Chr 11	Intergenic	
hsa-mir-371a	miR-371a-3p	**AAGUGCCG**CCAUCUUUUGAGUGU	Chr 19	Intergenic	miR-371~373 cluster
hsa-mir-371b	miR-371b-3p	**AAGUGCCC**CCACAGUUUGAGUGC			
**hsa-mir-372**	**miR-372-3p**	**AAAGUGCU**GCGACAUUUGAGCGU			
**hsa-mir-373**	**miR-373-3p**	**GAAGUGCU**UCGAUUUUGGGGUGU			
hsa-mir-512	miR-512-3p	**AAGUGCUG**UCAUAGCUGAGGUC	Chr 19	Intergenic	C19MC
hsa-mir-519a	miR-519a-3p	**AAAGUGCA**UCCUUUUAGAGUGU			
hsa-mir-519b	miR-519b-3p	**AAAGUGCA**UCCUUUUAGAGGUU			
**hsa-mir-519c**	**miR-519c-3p**	**AAAGUGCA**UCUUUUUAGAGGAU			
hsa-mir-519d	miR-519d-3p	**CAAAGUGC**CUCCCUUUAGAGUG			
hsa-mir-519e	miR-519e-3p	**AAGUGCCU**CCUUUUAGAGUGUU			
hsa-mir-520a	miR-520a-3p	**AAAGUGCU**UCCCUUUGGACUGU			
hsa-mir-520b	miR-520b	**AAAGUGCU**UCCUUUUAGAGGG			
**hsa-mir-520c**	**miR-520c-3p**	**AAAGUGCU**UCCUUUUAGAGGGU			
hsa-mir-520d	miR-520d-3p	**AAAGUGCU**UCUCUUUGGUGGGU			
hsa-mir-520e	miR-520e	**AAAGUGCU**UCCUUUUUGAGGG			
hsa-mir-520f	miR-520f-3p	**AAGUGCUU**CCUUUUAGAGGGUU			
hsa-mir-526b	miR-526b-3p	**GAAAGUGC**UUCCUUUUAGAGGC			

oncomotif

Supplementary Figure 2. Human microRNAs with AAGUGC-motif (oncomotif) in seed-sequence

An analysis of the human miRNA seed sequences resulted in the identification of 28 different miRNAs with an AAGUGC-motif (red), here referred to as oncomotif, in their seed sequence. Mature miRNA sequences were retrieved from miRBase 21, seed sequences (5' nucleotides 1-8) are indicated in bold. The 28 different miRNAs are expressed from 7 different genomic loci. All AAGUGC-miRNAs except hsa-mir-302e are present in genomic clusters with other AAGUGC-miRNAs. Two of these miRNA clusters (miR-17~92 and miR-106b~25) are located in an intron of a so called miRNA host gene (*MIR17HG* and *MCM7* respectively). All other AAGUGC-miRNAs are located in intergenic regions. Indicated in green are the four miRNAs identified in the functional genomics screen. These 28 miRNAs are here referred to as oncomotif-miRNAs.

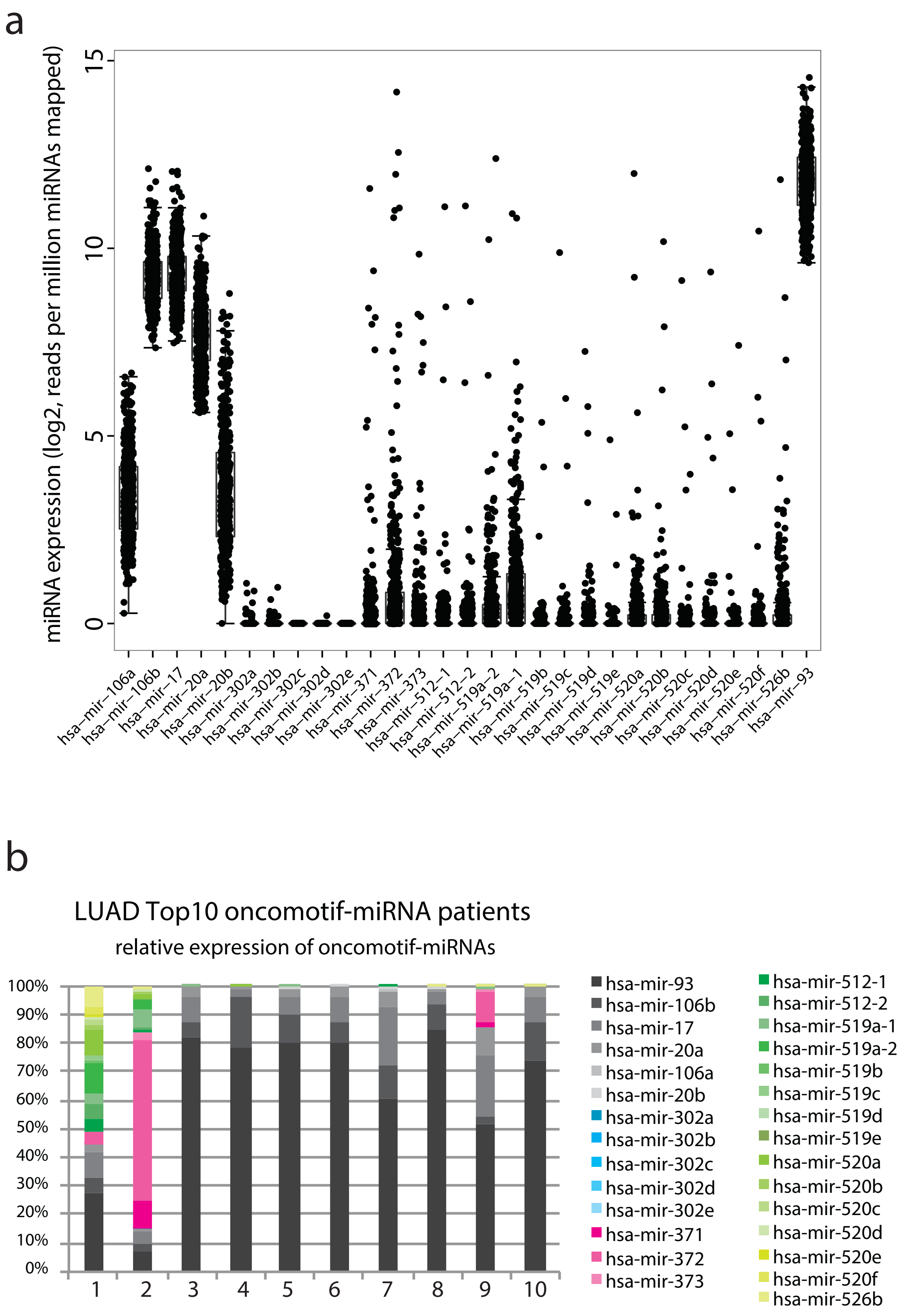

Supplementary Figure 3. Legend on next page

Supplementary Figure 3. Oncomotif-miRNA expression in lung adenocarcinoma

a. Boxplot showing the expression of individual oncomotif-miRNAs in 445 cases of lung adenocarcinoma (data retrieved from TCGA LUAD). The highest median expression is seen for hsa-mir-93, followed by hsa-mir-17 and hsa-mir-106b. Sporadic high expression is seen for oncomotif-miRNAs in the miR-371~373 cluster as well as the C19MC cluster.

b. Bar chart showing the relative contribution of different oncomotif-miRNAs to the sum of all oncomotif-miRNAs in the top10 oncomotif-miRNA expressing lung adenocarcinoma cases. In most cases hsa-mir-93 is the dominating miRNA, but occasionally expression from other loci is dominating as seen for the two tumors with the highest oncomotif-miRNA expression.

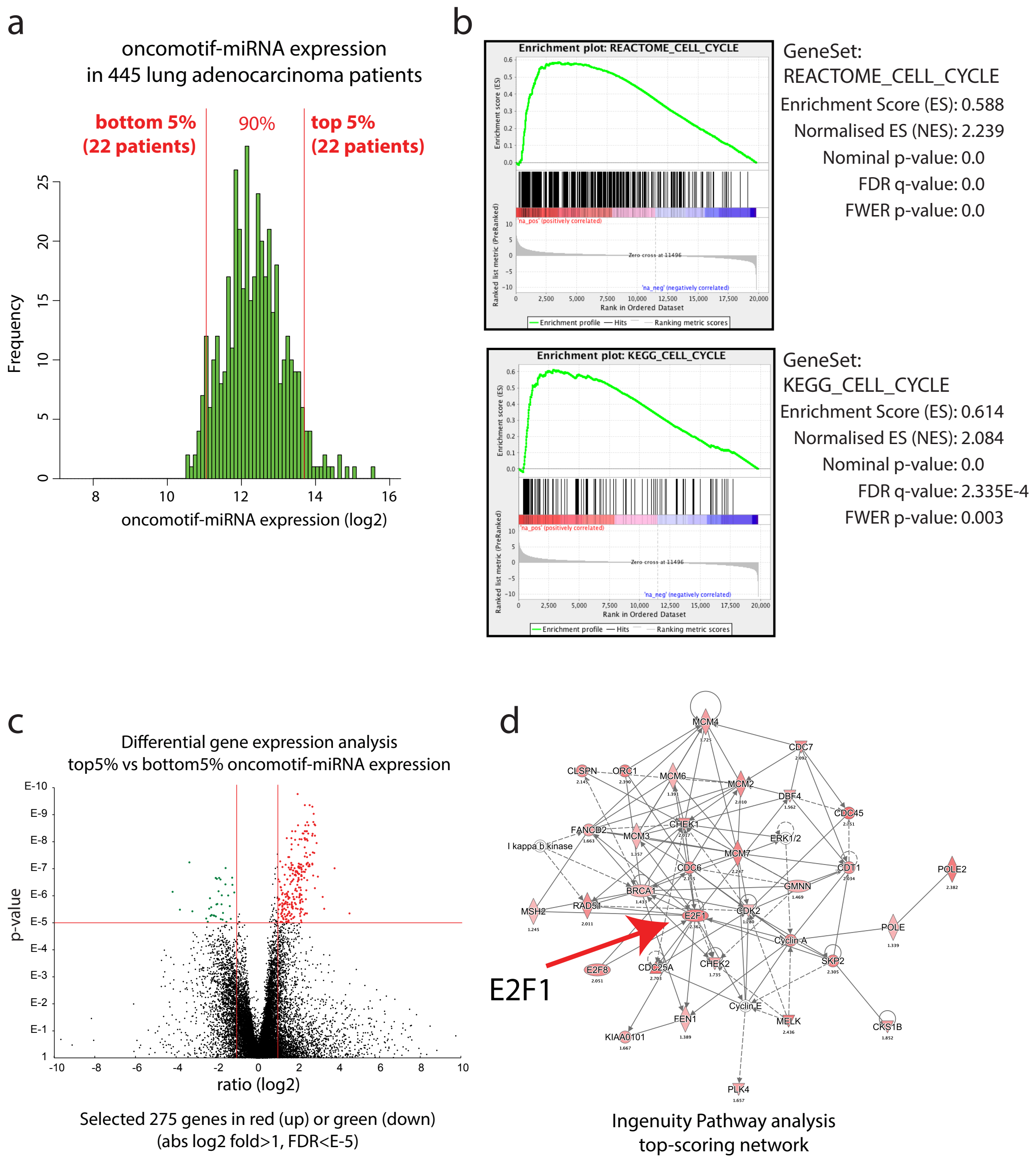

Supplementary Figure 4. Differential expression analysis between high and low oncomotif-miRNA expressing lung adenocarcinoma

a. Histogram showing the distribution of oncomotif-miRNA expression in lung adenocarcinoma (LUAD, TCGA). Oncomotif-miRNA expression is defined as the sum of reads from all individual oncomotif-miRNAs and expressed as reads per million microRNA reads mapped (log2). Differential expression analysis was performed between tumors with high or low oncomotif-miRNA expression (top vs. bottom 5 percentiles of 445 cases).

b. Gene set enrichment analysis (GSEA) plots for two top ranking cell cycle related gene sets showing significant enrichment in genes with higher expression in oncomotif-miRNA high LUAD cases.

c. Vulcano plot showing the results of differential expression analysis as defined in **a.** 275 genes with a two-fold difference between high and low oncomotif-miRNA LUAD cases (FDR p-value<E-5) were defined as oncomotif-miRNA signature genes. Legend continued on next page.

d. Ingenuity pathway analysis of the 275 oncomotif-miRNA signature genes resulted in a top-ranking cell cycle related network centered around E2F1.

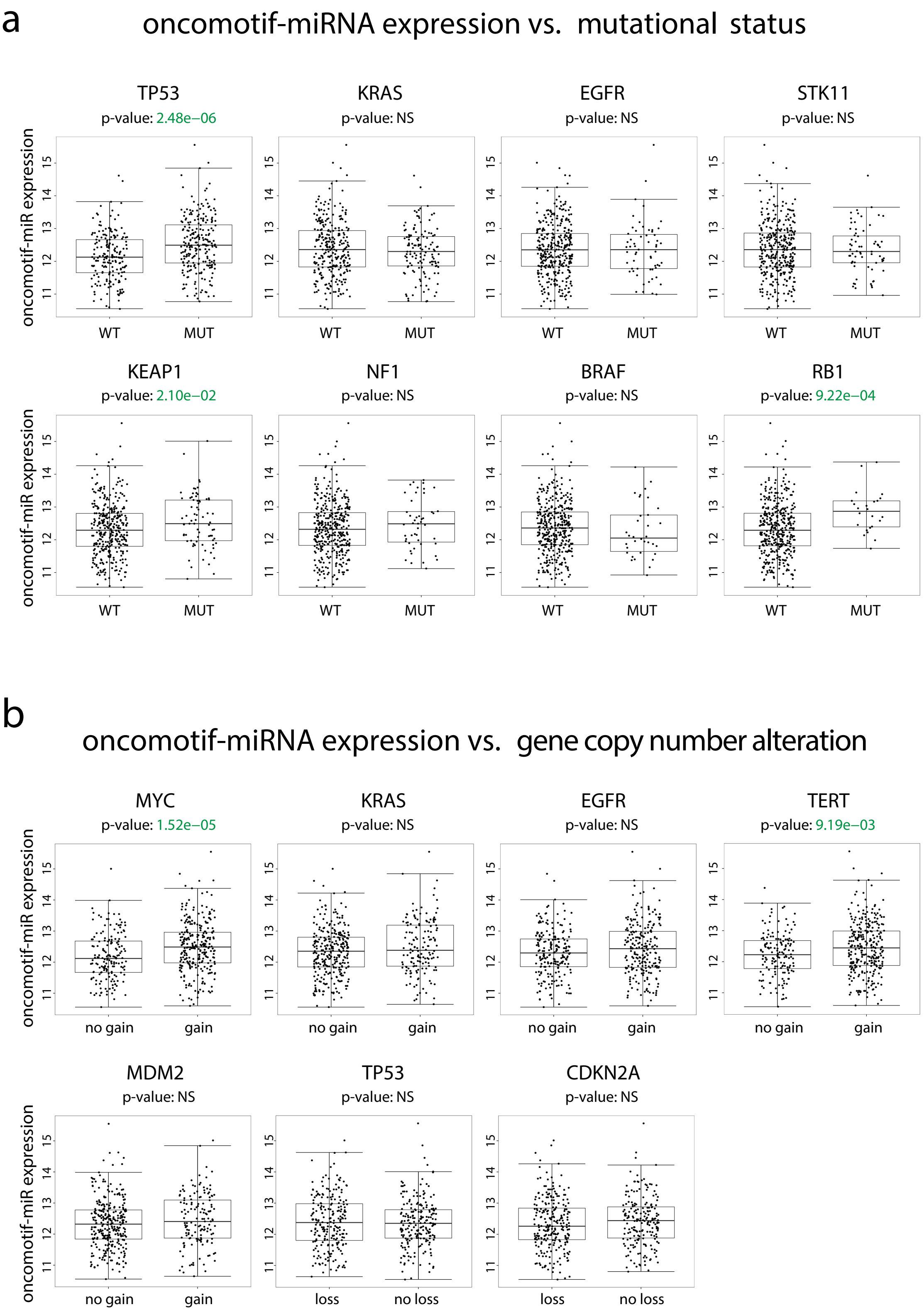

Supplementary Figure 5. Legend on next page

c

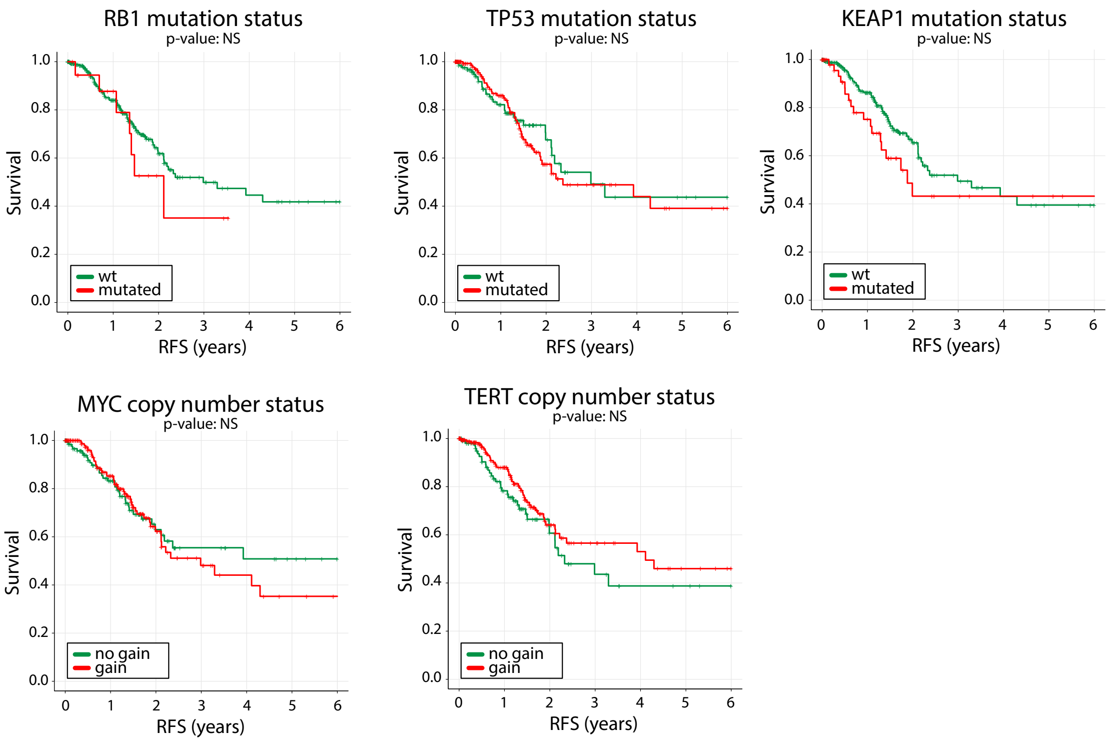

d

Hazard Ratio (univariate)

	HR	95% CI	p.val	No.
oncomotif-miRNA high cluster	1.72	1.09-2.72	1.97e-02	445
RB1 mut	1.42	0.65-3.08	NS	416
TP53 mut	1.06	0.68-1.66	NS	416
KEAP1 mut	1.55	0.92-2.59	NS	416
MYC cnv	1.10	0.71-1.70	NS	441
TERT cnv	0.72	0.46-1.11	NS	441

Hazard Ratio (multivariate)

	HR	95% CI	p.val	No.
oncomotif-miRNA high cluster	2.06	1.21-3.51	8.14e-03	413
RB1 mut	1.25	0.56-2.81	NS	413
TP53 mut	0.82	0.47-1.45	NS	413
KEAP1 mut	1.38	0.81-2.36	NS	413
MYC gain	0.97	0.71-1.33	NS	413
TERT gain	0.89	0.64-1.24	NS	413

Supplementary Figure 5. Oncomotif-miRNA expression and common genetic alterations in lung adenocarcinoma

a. Boxplots showing the expression of oncomotif-miRNAs in relation to mutation status of genes commonly mutated in lung adenocarcinoma (LUAD, TCGA). **b.** Boxplots showing the expression of oncomotif-miRNAs in relation to copy number of genes commonly showing copy-number variation in lung adenocarcinoma (LUAD, TCGA). **c.** Kaplan–Meier plots of relapse free survival (truncated at 6 years) for genetic alterations significantly associated with oncomotif-miRNA expression (**Supplementary Figure 5a** and **b,** indicated in green). **d.** Univariate and multivariate survival analyses by Cox proportional hazard regression for genetic alterations associated with oncomotif-miRNA expression. Number of TCGA samples included in each analysis depend on the availability of data from TCGA. Throughout the figure NS indicate p.values>0.05.

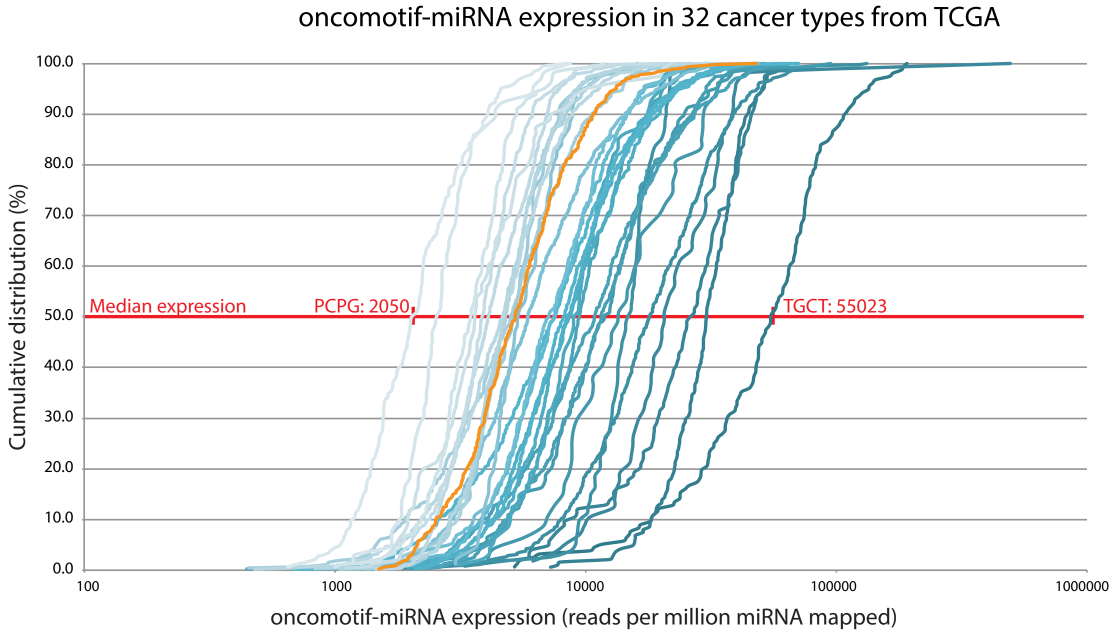

Decreasing median oncomotif-miRNA expression

TCGA Code	# samples	Description
TGCT	156	Testicular Germ Cell Tumor
LAML	188	AML
THYM	124	Thymoma
UCS	57	Uterine Carcinosarcoma
SKCM	451	Skin Cutaneous Melanoma
UVM	80	Uveal Melanoma
DLBC	47	Lymphoid Neoplasm DLBCL
ESCA	185	Esophageal carcinoma
LUSC	343	Lung SCC
BLCA	410	Bladder Urothelial Carcinoma
CESC	310	Cervical SCC and endocervical AC
READ	94	Rectum adenocarcinoma
UCEC	412	Uterine Corpus Endometrial Carcinoma
HNSC	488	Head and Neck SCC
LIHC	374	Liver hepatocellular carcinoma
STAD	396	Stomach AC
OV	493	Ovarian serous cystadenocarcinoma
COAD	247	Colon AC
BRCA	766	Breast invasive carcinoma
MESO	87	Mesothelioma
LUAD	452	Lung AC
CHOL	36	Cholangiocarcinoma
SARC	263	Sarcoma
LGG	526	Brain Lower Grade Glioma
KIRP	292	Kidney renal papillary cell carcinoma
PRAD	495	Prostate AC
THCA	514	Thyroid carcinoma
KIRC	255	Kidney renal clear cell carcinoma
ACC	80	Adrenocortical carcinoma
PAAD	179	Pancreatic AC
KICH	66	Kidney Chromophobe
PCPG	184	Pheochromocytoma and Paraganglioma

Supplementary Figure 6. Legend on next page

Supplementary Figure 6. Oncomotif-miRNA expression in 32 cancer types
Cumulative plot showing the distribution of oncomotif-miRNA expression within and across 32 cancers types (data retrieved from TCGA). The highest oncomotif-miRNA expression was seen in testicular germ cell tumor (TGCT, median oncomotif-miRNA expression 55,023), and the lowest in pheochromocytoma and paraganglioma (PCPG, median oncomotif-miRNA expression 2,050). Below the plot are the 32 different cancer types arranged from top to bottom according to the median oncomotif-miRNA expression. Indicated is also the number of cases analyzed for each cancer type.

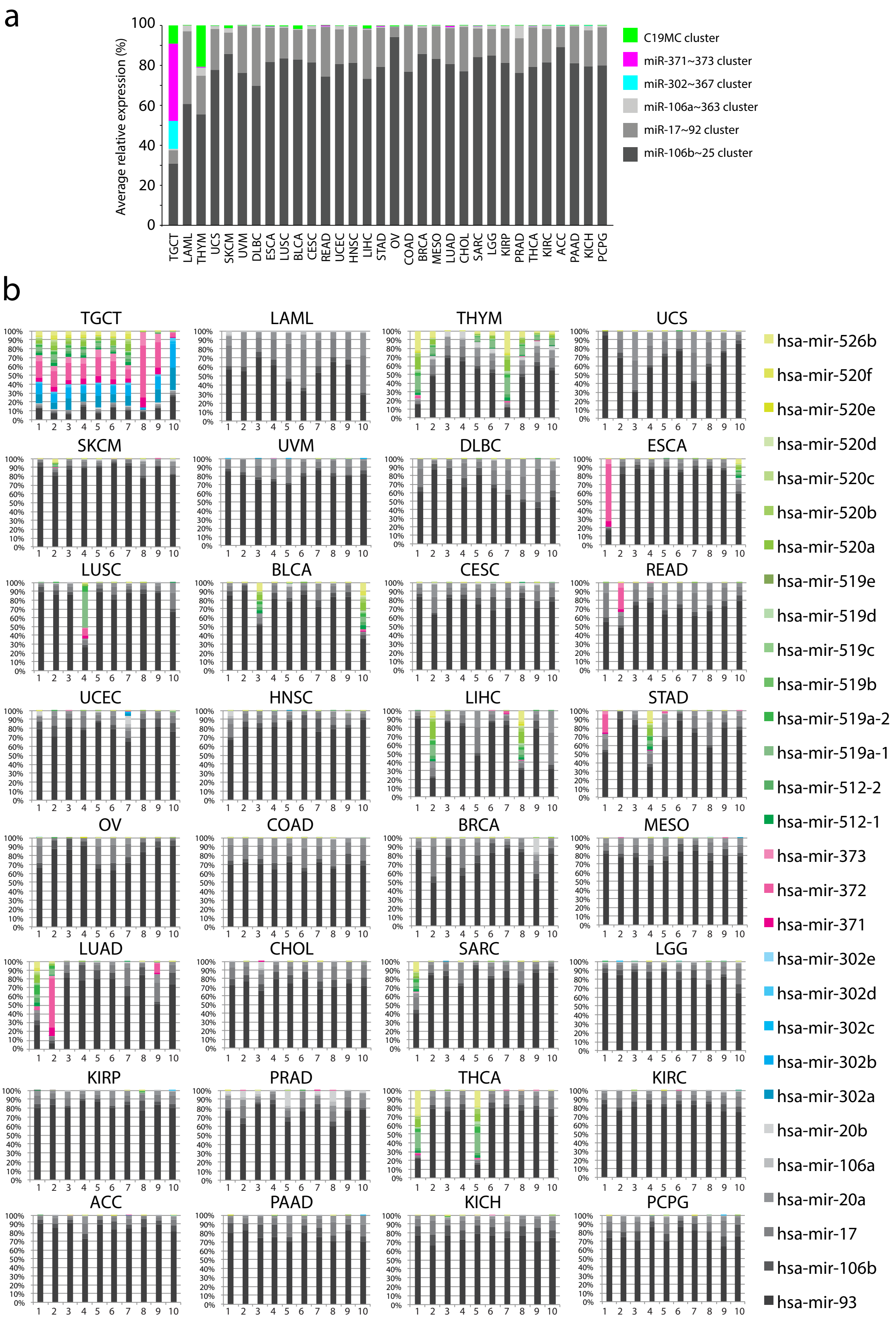

Supplementary Figure 7. Legend on next page

Supplementary Figure 7. Relative expression of different oncomotif-miRNAs in 32 cancer types. a. Bar chart showing the average relative contribution of different miRNAs (grouped by genomic clusters) to the oncomotif-miRNA expression in 32 different cancer types.

b. Bar chart showing the relative contribution of different miRNAs to the oncomotif-miRNA expression in the top10 expressing cases for each cancer type.

As shown in both **a** and **b**, hsa-miR-93 is the dominating oncomotif-miRNA in most cases with sporadic high expression of other miRNAs. Notable exceptions are testicular germ cell cancer (TGCT) with generally high expression of oncomotif-miRNAs from the miR-371~373 , C19MC and miR-302~367 clusters, as well as thymoma (THYM) that show common expression of miRNAs from the C19MC cluster.

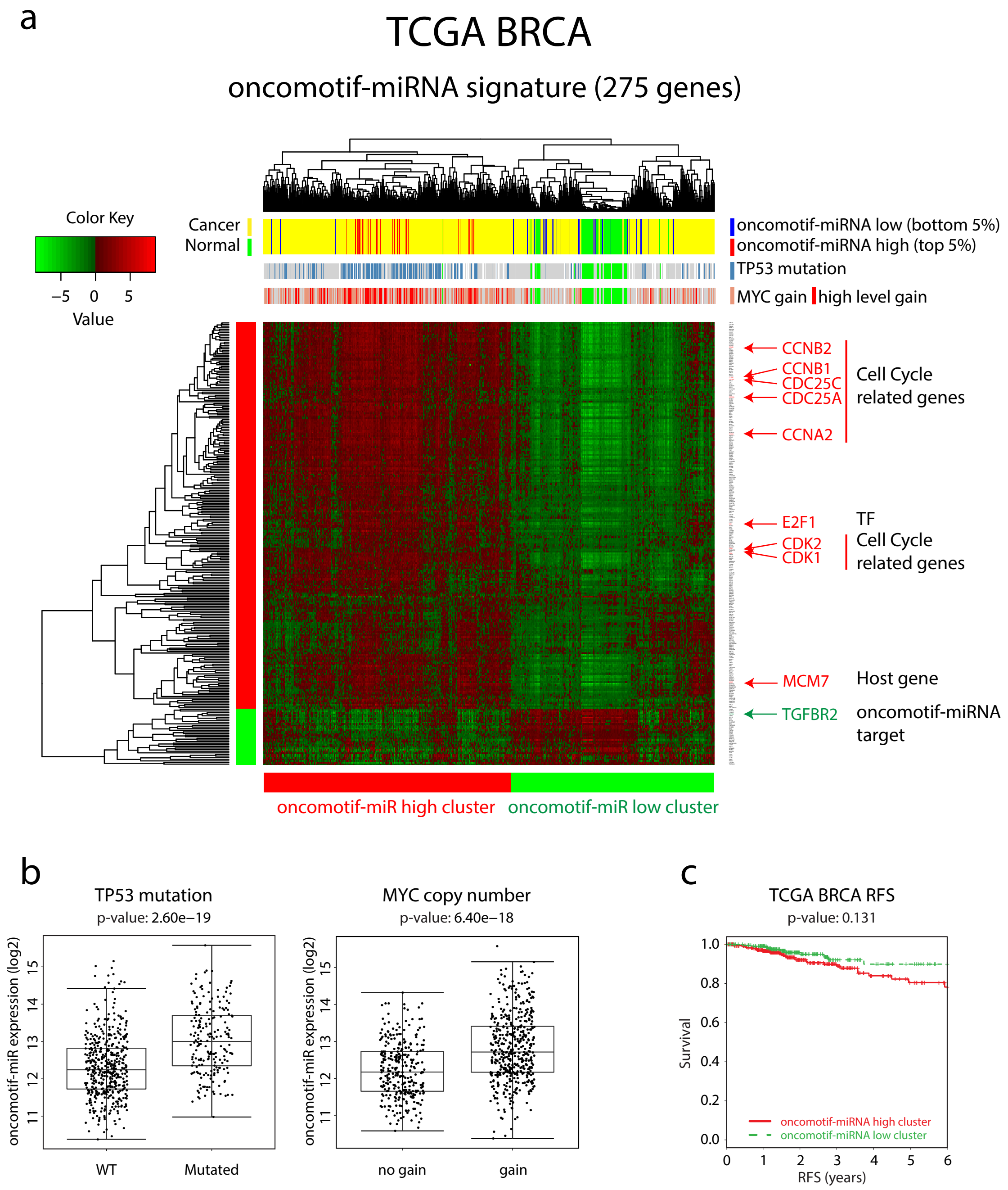

Supplementary Figure 8. Legend on next page

Supplementary Figure 8. Cluster analysis of breast cancer based on oncomotif-miRNA signature genes.

a. Heatmap showing the results of hierarchical clustering (patients horizontally and genes vertically) of 762 breast invasive carcinoma samples and 87 normal breast epithelium samples (BRCA, TCGA) based on the expression of the 275 oncomotif-miRNA signature genes (see **supplementary figure 4** for details). Heatmap colors represent mean centered log2 fpkm values. Inserts, from top to bottom, indicate; cancer (yellow), normal (green), oncomotif-miRNA high cancer (top5%, red) and low cancer (bottom5%, blue); TP53 mutated (blue) or TP53 wt (grey); MYC gain (light red), MYC high-level gain (red) or no gain (grey). Indicated on the right are a few genes with increased expression (red, oncomotif-miRNA transcription factor, oncomotif-miRNA host gene and cell cycle related genes) or decreased expression (green, oncomotif-miRNA target) in the oncomotif-miRNA high compared to low clusters.

b. Boxplots showing the expression of oncomotif-miRNAs in relation to *TP53* mutation and *MYC* amplification in breast invasive carcinoma (BRCA, TCGA).

c. Kaplan–Meier plot of relapse free survival (truncated at 6 years) for the oncomotif-miRNA high cluster patients (red, n=466) and low cluster patients (green, n=296).
